# TAL Effectors Specificity Stems from Negative Discrimination

**DOI:** 10.1371/journal.pone.0080261

**Published:** 2013-11-25

**Authors:** Basile I. M. Wicky, Marco Stenta, Matteo Dal Peraro

**Affiliations:** 1 Laboratory for Biomolecular Modeling, Institute of Bioengineering, School of Life Sciences, École Polytechnique Fédérale de Lausanne (EPFL), Lausanne, Switzerland; 2 Swiss Institute of Bioinformatics (SIB), Lausanne, Switzerland; German Research School for Simulation Science, Germany

## Abstract

Transcription Activator-Like (TAL) effectors are DNA-binding proteins secreted by phytopathogenic bacteria that interfere with native cellular functions by binding to plant DNA promoters. The key element of their architecture is a domain of tandem-repeats with almost identical sequences. Most of the polymorphism is located at two consecutive amino acids termed Repeat Variable Diresidue (RVD). The discovery of a direct link between the RVD composition and the targeted nucleotide allowed the design of TAL-derived DNA-binding tools with programmable specificities that revolutionized the field of genome engineering. Despite structural data, the molecular origins of this specificity as well as the recognition mechanism have remained unclear. Molecular simulations of the recent crystal structures suggest that most of the protein-DNA binding energy originates from non-specific interactions between the DNA backbone and non-variable residues, while RVDs contributions are negligible. Based on dynamical and energetic considerations we postulate that, while the first RVD residue promotes helix breaks – allowing folding of TAL as a DNA-wrapping super-helix – the second provides specificity through a negative discrimination of matches. Furthermore, we propose a simple pharmacophore-like model for the rationalization of RVD-DNA interactions and the interpretation of experimental findings concerning shared affinities and binding efficiencies. The explanatory paradigm presented herein provides a better comprehension of this elegant architecture and we hope will allow for improved designs of TAL-derived biotechnological tools.

## Introduction

**Figure 1:**
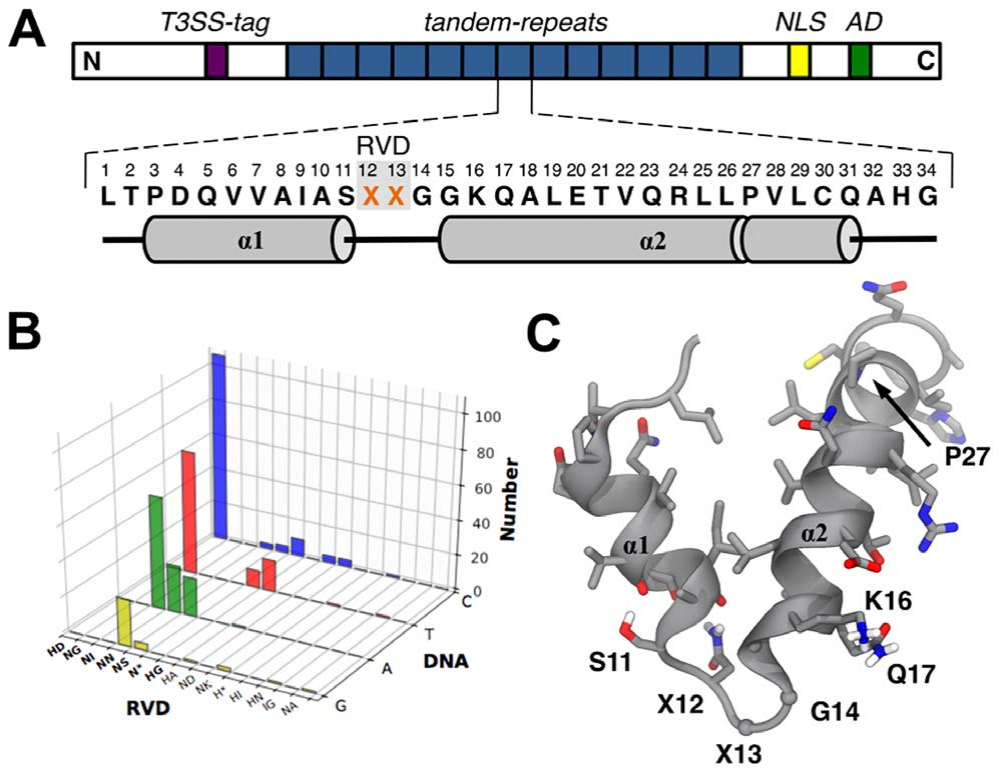
TAL topology and RVD-to-DNA code. (**A**) Sequence of a TAL protein: type III secretion system tag (T3SS-tag, violet), tandem-repeat domain (blue), nuclear localization signal (NLS, yellow) and acidic transcriptional activation domain (AD, green). The amino acid sequence of a single repeat is shown, highlighting the RVD region (X12 and X13). The secondary structure is reported underneath; the kink induced by P27 is represented as a break in the α2 rod. (**B**) Natural occurrence of the known RVDs is reported together with the targeted DNA base to highlight RVD selectivity [4]. While some RVDs target only a single base (*e.g.* HD and ND), others have shared affinities (*e.g.* NN and N*). (**C**) Representative structure of one repeat as extracted from the 3V6T structure; relevant molecular information is highlighted.

TAL (Transcription Activator-Like) effectors are proteins secreted by phytopathogenic Gram-negative bacteria from the *Xanthomonas* genus [1], responsible for the infection of more than 200 different plant families, including many crops [2]. TAL effectors are injected directly into the host plant cells *via* Type III Secretion System (T3SS) and, after localization to the nucleus, specifically bind to DNA sequences, thus interfering with native cellular functions and supporting infection [1]. Endogenous TAL proteins are composed of an N-terminal translocation signal necessary for T3SS injection, a C-terminal nuclear localization signal (NLS) domain and an acidic activation domain (AD), both important eukaryotic motifs (Figure 1A) [2], [3]. The central domain is composed of a variable number of tandem- repeats, ranging from 1.5 to 33.5, with 17.5 being the most abundant [2]. The number of repeats correlates with the number of base pairs in the targeted sequence and each repeat is composed of an almost invariable stretch of 34 amino acids [2]. Polymorphism among repeats occurs predominantly at positions 4, 12, 13 and 32 (internal numbering), with positions 12 and 13 being by far the most variable, and accordingly termed Repeat Variable Diresidue (RVD) [2], [4].

TAL systems recently gained wider attention after a direct relationship between the RVD type and the targeted DNA base was established by both experimental [5] and bioinformatics methods [4]. The DNA specificity of RVDs is univocally determined for only some amino acid combinations, while in many cases affinity for multiple nucleobases is observed [4]. Position 12 was found to be either H or N, and position 13 being one of D, G, I, N, S, A, K or missing altogether (*). The combination of these amino acids accounts for about 95% of all known RVDs (Figure 1B) [4]. The discovery of this code opened the way to unforeseen applications, far beyond the scope of plant cell manipulation. TAL-based tools established a new landmark in site-specific genome regulation and modifications. Fusion of TAL with activator or repressor domains has provided functional transcription factors with desired sequence specificities [5−7] and careful design has allowed orthogonal site-targeting [8]. Fusion with nucleases has yielded tools for site-specific double-strand break generation, for either gene knockout or user-defined *cassette* insertion [9−12]. TAL nucleases (TALENs) have been shown to work in a wide range of organisms, from yeast to mammalian cells [7], . If geneticists had ever dreamed of a modular and predictable DNA-targeting tool, it probably would have resembled TAL.

**Figure 2:**
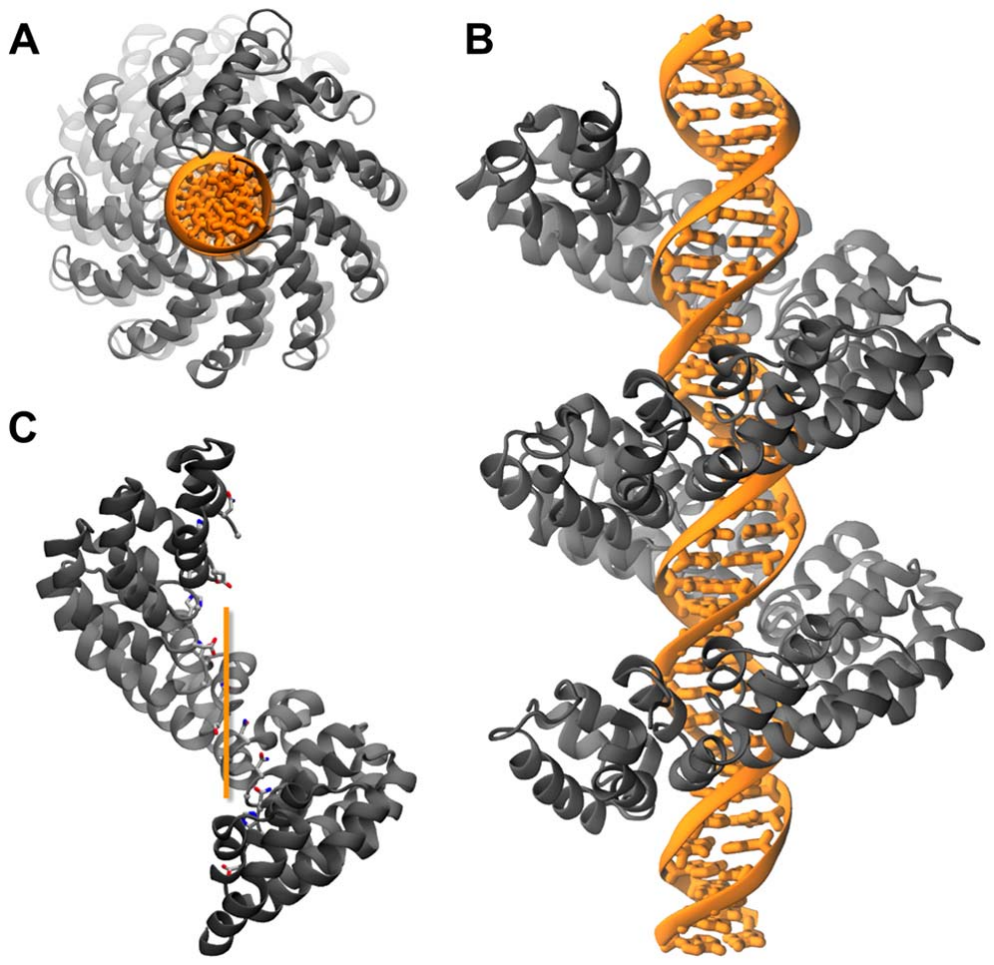
TAL repeats architecture. (**A**) Top view of bound TAL along the DNA axis showing the N-terminus (structure from 3UGM). (**B**) Side view of bound TAL displayed from the N-terminus (bottom) to the C-terminus (top) (structure from 3UGM). (**C**) TAL without DNA and the RVDs explicitly depicted (laying on the inner-side of the super-helix). The orange line represents the DNA axis. The protein is orientated from N-terminus (bottom) to C-terminus (top). A larger pitch compared to the bound structure is clearly observable (structure from 3V6P). Protein: grey; DNA: orange.

The understanding of the structural features of TAL effectors in general, and their relationships to DNA-binding in particular, are essential steps towards effective protein engineering for tailored biotechnological applications. Following the first structural NMR data of a single TAL repeat [17], both DNA-bound and free forms of TAL proteins have been crystallized. Mak *et al.* obtained the structure of PthXo1, a naturally-occurring 23.5 repeats TAL bound to its corresponding DNA target, at 3 Å resolution [18] (PDB code: 3UGM), while Deng *et al.* solved the structure of a designed 11.5 repeats TAL system in both bound and unbound states with resolutions of 1.85 Å and 2.5 Å respectively [19] (PDB codes: 3V6T, 3V6P). All structures showed the same overall architecture, with TAL forming a right-handed, highly symmetrical super-helix wrapped around a regular B-DNA double-strand (Figure 2). Each repeat is composed of two anti-parallel helices, α1 and α2, the latter being roughly twice as long as the former and possessing a kink at the position of residue P27 (Figure 1A/C). The RVD, located on the loop linking α1 to α2, interacts with the major groove of the DNA sense strand. Residues K16 and Q17, close to the RVD loop, also contribute to DNA binding through non-specific polar and charged interactions with the DNA backbone. Although each RVD consists of two amino acids, only the residue at position 13 seems to directly interact with DNA in the crystal structures. It has been suggested that position 12 influences the orientation of the side-chain of residues at position 13 through non-direct interactions such as water-bridges and RVD loop stabilisation [19]. The crystallographic structures provided a molecular perspective on the process of DNA recognition mediated by RVDs, highlighting in particular the interaction of D13 with the amino group of cytosine, the van der Waals contact of G13 with the methyl group of thymine, the electrostatic interaction of N13 with nitrogen 7 of purine bases and the van der Waals interactions of I13 with either adenine or cytosine [18], [19]. The molecular basis of the different interactions has been reviewed on the basis of the crystallographic structures [20], [21].

Although those structures have provided a much clearer understanding of DNA recognition by TALs, some aspects of this unique mechanism still remain elusive. In particular, the source of high specificity in RVD-DNA base recognition – despite limited interactions – and the grounds for both RVD compositions and occurrences are still unclear. Moreover, the structural roles of the other residues and their importance to protein function have not been addressed yet. Furthermore, although the RVD-to-DNA code has been shown to work well in most cases, some combinations have proven sub- or non-functional, raising the issue of context dependence [12], [15], [22]. Finally, the role of DNA methylation on TAL-DNA binding [23] should be extended to other RVDs compatible with methylated DNA. Addressing these points will prove essential for achieving full protein-engineering capabilities and effectively design new TAL systems with improved DNA-binding abilities and specificities. In this report, we sought to address these questions by using molecular dynamics (MD) simulations to complement the picture obtained from crystallographic structures with dynamical and energetic information.

## Materials and Methods

### Molecular dynamics simulations

The available crystal structures of both free (PDB code: 3V6P) [19] and DNA-bound (PDB codes: 3UGM, 3V6T) [18], [19] TAL effectors were used to build model systems in the framework of classical molecular mechanics [24]. Molecular dynamics simulations using NAMD 2.8 [25] with the AMBER ff99brsc force field [26], an explicit solvent model (TIP3P [27]) and periodic boundary conditions were performed on the constructed systems to complement the information obtained from the X-ray structures [28] (Table 1). First, geometry optimisations with 1000 steps of energy minimization using a conjugate gradient were performed in order to relax the systems. Dynamics started by an equilibration in the NVT ensemble at 100 K, followed by a gentle rise of the temperature to 300 K in 300 ps and a further NVT (300 K) equilibration phase. All those steps were performed with a harmonic constrain applied to the heavy atoms (*i.e.* not H) of the protein and DNA. Productions were run in the NPT ensemble (1 atm, 300 K) with all atoms free. Langevin dynamics was used to enforce ensemble parameters. A 12 Å cutoff distance was defined for short-range interactions, while the Particle Mesh Ewald summation method was used to compute long-range interactions. The RATTLE algorithm was used to treat covalent bonds involving hydrogen atoms in order to allow an integration step of 2 fs. Ions (Mg^2+^ and Cl^−^) were added to the systems to neutralize charges and obtain a final magnesium concentration mimicking physiological conditions (∼50 mM). The structural ensembles generated by MD simulations were used to establish statistics on the conformational states accessible to the systems. This information was used to highlight the structural importance of certain residues in the protein architecture and for the DNA recognition mechanism. Full details about system set-ups and MD simulation protocols are reported in Methods S1.

**Table 1:** Simulation details for each system.

	PDB	Selected System	RVD His Protonation	Number of Atoms	Box Size [Å^3^]	Simulation Length [ns]
**TAL[22.5]/P1**	3UGM	*Protein*: 192 to 1048 (chain A); *DNA*: −6 to 29 (chain B) and 1 to 36 (chain C)	Nδ	158675	106×151×107	53
**TAL[22.5]/P2**	3UGM	*Protein*: 192 to 1048 (chain A); *DNA*: −6 to 29 (chain B) and 1 to 36 (chain C)	Nε	158675	106×151×107	61
**TAL[11.5]/P1**	3V6T	*Protein*: 231 to 721 (chain A); *DNA*: −2 to 14 (chain I) and −14 to 2 (chain J)	Nδ	107599	101×102×112	127
**TAL[11.5]/P3**	3V6T	*Protein*: 231 to 721 (chain A); *DNA*: −2 to 14 (chain I) and −14 to 2 (chain J)	Nδ + Nε (charged)	107618	101×102×112	57
**TAL[11.5]/P4**	3V6T	*Protein*: 231 to 721 (chain A); *DNA*: −2 to 14 (chain I) and −14 to 2 (chain J)	Nδ + Nε (charged and ring flipped)	107618	101×102×112	42
**TAL[11.5]/P1-apo**	3V6T	*Protein*: 231 to 721 (chain A); *DNA*: no DNA	Nδ	107635	101×102×112	51
**TAL** [**10**] **/P1-apo**	3V6P	*Protein*: 303 to 675 (chain A); *DNA*: no DNA	Nδ	95742	108×96×99	56

### Binding energy calculations

To further investigate TAL specificity, energy decompositions of pairwise interactions were performed using MM/GBSA (Molecular Mechanics/Generalized Born Surface Area) and MM/PBSA (Molecular Mechanics/Poisson Boltzmann Surface Area) implicit solvent calculations in order to identify the hot spots at the protein-DNA interface and the stabilizing interactions within TAL [29−31]. All calculations were performed with the parallelized version of MM/PB(GB)SA implemented in the Amber 11 suite of program [32] using the standard single-trajectory approach. Entropy contributions were not taken into account. Each MM/PB(GB)SA calculation was performed on 500 snapshots evenly taken from the last 30 ns of the corresponding equilibrated simulations (one snapshot every 6 ps of MD simulation). Furthermore, a non-standard procedure involving MM/GBSA and MM/PBSA calculations was performed in order to qualitatively estimate the dependence of binding energy on the number of TAL repeats wrapping the DNA double-strand (*cf.* Methods S1 and Figure S12).

### Quantum mechanical calculations

Quantum mechanical calculations were performed to obtain the molecular electrostatic potential (MEP) of DNA bases. The Gaussian 09 suite of program [33] was used to perform geometry optimisation and MEP calculations of nucleobases at the B3LYP/6-31+G*//B3LYP/6-31+G* level. Methyl caps were added on the N9 of purine bases and N1 of pyrimidine bases.

## Results and Discussion

### DNA templates TAL architecture and rigidifies the RVD motif

**Figure 3:**
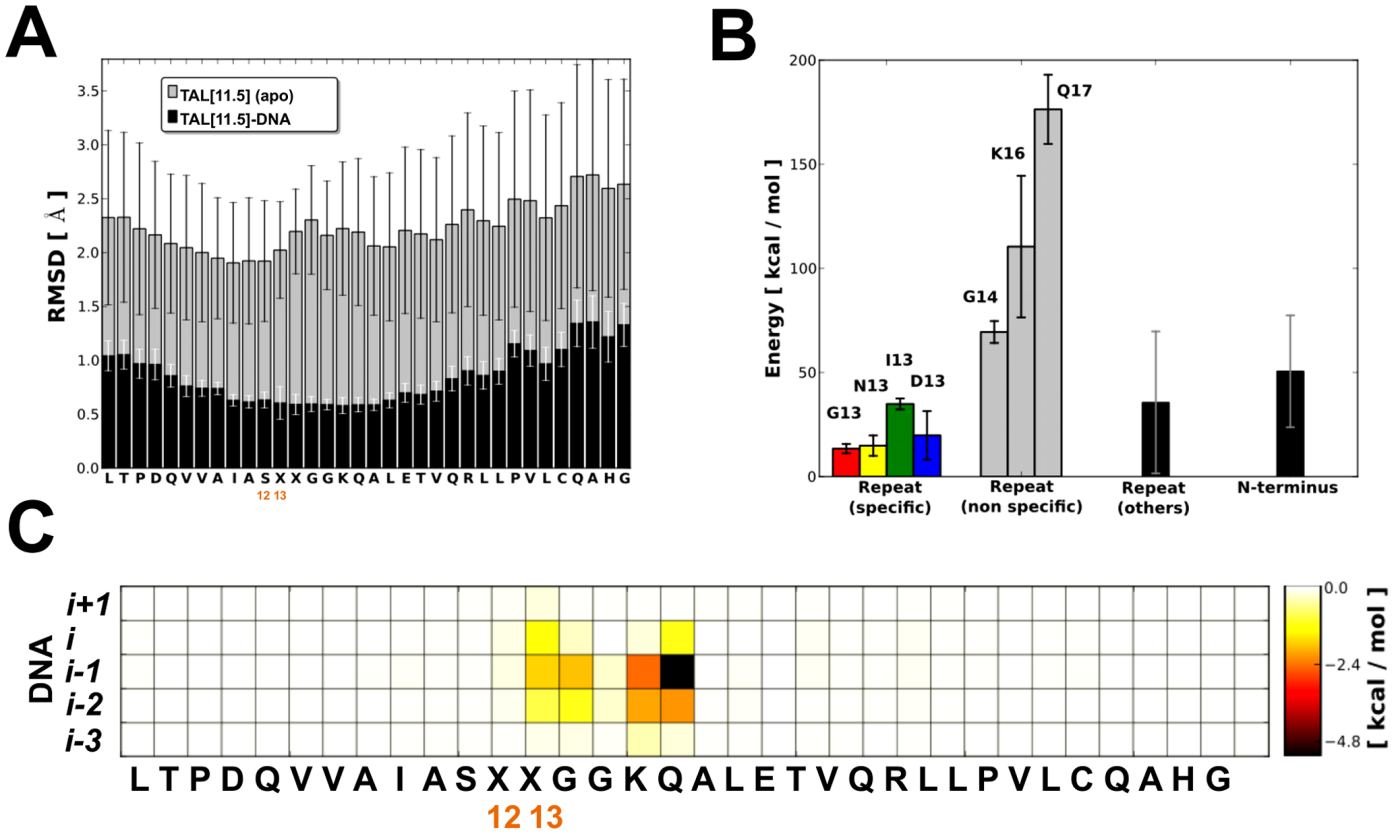
Structural and energetic features of TAL-DNA interaction. (**A**) Mean residue fluctuation (RMSD) computed for the DNA-bound and *apo* states of the 11.5-repeats TAL system (TAL[11.5]/P1 and TAL[11.5]/P1-apo); averages are performed over all the repeats; bars represent standard deviations. The same trend is observed for all simulated systems (*cf.* Figure S5). (**B**) Contribution to the total DNA-binding energy from different sections of TAL subdivided by type and calculated on the DNA-bound 22.5-repeat TAL system (TAL[22.5]/P1) using MM/GBSA (Number of residues contributing to each type: G13 = 6, N13 = 2, I13 = 7, D13 = 5, G14/K16/Q17 = 20, others = 600, N-terminus = 97). Repeats containing a deletion at position X13 have been excluded from the statistics. (**C**) Per repeat mean energy contribution to the total DNA-binding energy; averages are performed over all the repeats of the DNA-bound 22.5-repeat TAL system (TAL[22.5]/P1). Repeats containing a deletion at X13 position have been excluded from the statistics. The complete binding energy profile is reported in Figure S9.

All simulated TAL-DNA systems showed comparable root mean square deviations and positional fluctuations; between 4.0 and 4.8 Å for the large systems, and between 1.9 and 2.5 Å for the small systems. Differences among system categories (TAL[22.5] or TAL[11.5]) are not statistically significant (*cf.* Figures S1-S3 **a**, **b** and **c**, **e**, **f**). All systems are stable with conservation of secondary structure within the simulated time-scales (Figure S4). For the DNA-bound systems, larger structural fluctuations were observed at the distorted termini (Figure S3). Within the tandem-repeat section, the largest fluctuations were found in the peripheral regions of each, while the innermost residues in contact with DNA (including the RVDs) showed a remarkable reduced mobility (Figure 3A and Figure S5). Interestingly, different structural fluctuations were observed for the sense (*i.e.* recognised by TAL) and anti-sense strands of DNA (see Figure S1 **a**, **b**, **c**, **e**, **f**). Those data are in accordance with the B-factors of the crystallographic structures.

In the absence of DNA, TAL proteins are more flexible (Figures S1-S3 **d**, **g**) and the RVD loops fluctuate as much as the outer region (Figure 3A and S5 **d**, **g**) – 2.1 +/− 0.5 Å when unbound *vs* 0.6 +/− 0.2 Å when bound; values are for the RVD loops of the small system – showing an overall stabilizing effect of the DNA double helix on TAL architecture. Moreover, when DNA is removed from the DNA-bound crystal structure 3V6T prior to simulation (TAL[11.5]/P1-apo), the protein rapidly stretches and the pitch approaches that of the unbound TAL 3V6P (Figure 2C). This dependence of the pitch value on the presence of DNA is consistent with the results of Murakami *et al.* [17], reporting that adjunction of dsDNA to TAL reduced its hydrodynamic radius. Taken together these results indicate that DNA acts: *i)* globally, as template in the structural rearrangement of TAL systems from a stretched to a packed structure and *ii)* locally, to stabilize and reduce the mobility of the RVD loops.

### TAL repeats are packed through stabilizing interactions

The analysis of the interaction energetics extracted from MD simulations revealed that most of the intra-protein stabilization is due to non-specific backbone-backbone interactions within α-helices (Figures S6-S7). This property is consistent with the large helical content of the protein and the requirements for secretion through the T3SS injectisome. According to recent studies on T3SS [34], protein effectors partially unfold to cross the narrow injectisome needle. Thus, TAL systems, composed by loosely connected α-helices, can cross the needle without significant loss of secondary structure. Specific side-chain interactions occur mostly at the transition between coil-regions and α-helices and between consecutive repeats (Figure S8). The interactions between the outer-coil and α1 (including T2 to A4/Q5 and Q5 to L1/T2) are energetically significant and stable over dynamics. Interaction of the hydroxyl group of S11 with the backbone carbonyl of V7 and interaction of an N-H from the X12 side-chain (first RVD residue; either asparagine or histidine) with the I9 backbone carbonyl represent significant energetics at the structural transition between α1 and the RVD loop. Surprisingly, the interaction of X12 with the backbone carbonyl of A8 reported in the crystal structure [19] was less significant from an energetic point of view (Figure S8). This interaction pattern suggests that the mentioned residues promote a structural transition from coil to helix and *vice versa*; an essential feature to attain the DNA-wrapping super-helix architecture characteristic of TAL proteins. Interactions of repeat *i* with its neighbours *i*-1 and *i*+1 are mainly constituted by K16(*i)*-Q17(*i-1*) and E20(*i)*-R24(*i-1*), forming a stable H-bond network linking together the TAL repeats in a regular and stable structure (Figures S8). Much less important are the interaction of H33(*i*) with Q23(*i+1*)/L26(*i+1*)/P27(*i+1*), all located at the kink generated by P27 (Figure S8). The presence of this conserved motif among repeats could suggest a pH-responsive mechanism for protein packing during secretion, with the strength of both intra- and inter-repeat interactions being modulated by the protonation state of histidine. However, further experimental evidences are required to address this proposition.

### RVDs marginally contribute to TAL-DNA interaction energetics

Detailed information about TAL-DNA interactions at the single-residue level can be extracted from the decomposition of pairwise interaction energies as calculated at the molecular mechanics level [24] and averaged over equilibrated MD trajectories using an implicit solvent model (Figure 3B/C and Figures S9-S11). This approach has been successfully applied to investigate the energetics of protein-protein as well as protein-DNA interactions [35−39].

Strikingly, non-specific interactions between DNA backbone of the sense strand and residues G14, Q17 and K16 account for most of the overall binding energy of TAL proteins to DNA (Figure 3B/C). The N-terminal region also contributes significantly to the binding (Figure 3B and Figures S9-S11), consistent with the experimental finding that the N-terminus serves as a nucleation point for DNA wrapping after non-specific interactions with an upstream sequence [5], [40]. Unexpectedly, when considering the high specificities of TAL proteins for their targets, both RVD residues contribute only to a small fraction of the total DNA binding energy. The first position in each RVD (X12, either H or N) does not form any direct interaction with DNA, neither in the major-groove, nor with the backbone of the lagging strand (Figure 3C and Figures S9-S10), but instead forms a stable interaction with the carbonyl group of I9 (Figure S8). The presence of histidine residues at the first position of certain RVDs prompted us to investigate the effect of its different protonation states. However, no statistically significant differences on binding energetics and dynamical behaviours were observed (see Figures S1-S5 **a**, **b** and **c**, **e**, **f** and S11). The second position of each RVD (X13) accounts for a small, although significant, fraction of the remaining DNA-binding energy (Figure 3B/C). Not only does it interact with its cognate nucleobase, but it also makes a substantial interaction with the preceding DNA base (Figure 3C). As a matter of fact, on average, interaction between each *i^th^* RVD and the *i-1* nucleobase is stronger than the interaction with the *i^th^* base. This effect can be attributed to NG RVDs, which constitute the largest fraction of all the RVDs found in the 23.5-repeat structure (Figure S9). Results from electrophoretic mobility shift assays showed that NG was the RVD with the highest DNA-binding properties [41], thus correlating with our energetic data. Although unexpected, this observation can be explained by considering the stair-like arrangement of DNA bases. The same effect has been observed for another DNA-binding protein, where correlation has been made between binding energy and the molecular surface of nucleobases displayed to protein side-chains [42]. This phenomenon provides a rationale for context dependence of TAL DNA-binding. Indeed, depending on the combination of RVD and *i-1* DNA base, this extra interaction could either be favoured or disfavoured, in turn affecting the overall binding of TAL to DNA.

### The origin of TAL-DNA specificity

TAL systems have evolved to bind DNA sequences with high specificity. Selectivity is ensured by a central domain composed of a variable number of repeats, each responsible for recognizing a single base. The capability of TAL to bind the target sequence can be often impaired by disruption of only one RVD-base interaction [15]. Strikingly, only a small subset of the 20 natural amino acids is present in the naturally occurring RVDs, with about 95% of all known RVDs obtained from the combination of H, N, I, D, S, and G only. Both X-ray structures and our dynamical simulations indicate that there is neither interaction between the two RVD residues nor correlated motion. Thus, the amino acid populations of position 12 and 13 can be treated separately and their effect on DNA binding and base selectivity deconvoluted.

The first RVD position (X12) is almost always either H or N, the side-chain of either of them forming a stable interaction with the backbone carbonyl of I9; this promotes the helix break between α1 and α2 and allows the formation of the RVD loop in between. The low intrinsic α-helix propensities of N and H [43−45] also argue for X12 as a helix-breaker. This hypothesis is further supported by the strong interaction energetics of S11 with the backbone carbonyl of V7, further contributing to the formation of the RVD loop. This suggests a purely structural role for X12, unrelated with the DNA recognition process. The grounds for two different side-chains to perform a seemingly identical task remains elusive but the recurrent H12-D13 RVD motif could imply either a dependence to pH or a way to stabilize the negative charge of the aspartate (D13) in the strong electric field present close to the phosphodiester DNA backbone. However, no relevant differences in either dynamical features or DNA-binding energetics were observed between systems featuring different protonation states of the histidine side-chain (see Figures S1-S5 **a**, **b** and **c**, **e**, **f** and S11).

**Figure 4:**
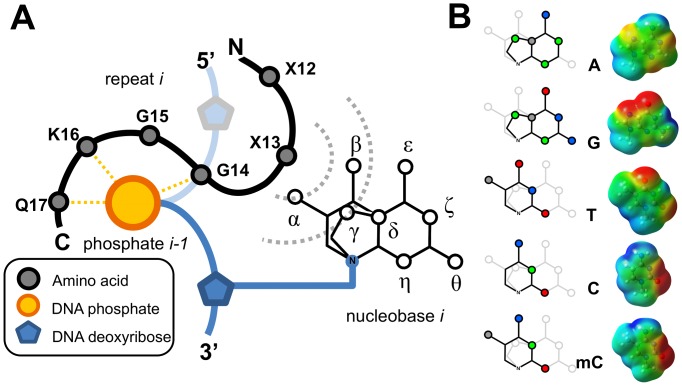
Schematic representation of the TAL-DNA interface. (**A**) Cartoon representation of one single repeat interacting with DNA. The *oxyanion clip* (G14, K16 and Q17) interacts with the phosphate group of the *(i-1)^th^* base, thus fixing the position of the X13 side-chain with respect to the *i^th^* base and freezing its structural fluctuation. Dashed circles indicate the interaction radii of different X13 residues, sorted by side-chain size: the inner circle corresponds to G13/*13, while the others are represented by the outer circle. Only loci α, β and γ are sampled by the side-chains of X13, resulting in incomplete molecular differentiation. (**B**) Pharmacophore-like model for the nucleobases discussed in the text (left). Dots represent sites with variable properties across nucleobases; colours are used to highlight the characteristics of the substituents: green for pyridine-like (H-bond acceptor) nitrogen atoms, blue for pyrrole-like and amine (H-bond donor) nitrogen atoms, grey for methyl groups, and red for carbonyl oxygen atoms. On the right, relative molecular electrostatic potential (MEP) maps for the corresponding nucleobases are reported. Calculations were performed at the QM level on methyl-capped purines (N9) and pyrimidines (N1) (red  =  −5.0 k_B_T/e, blue  =  5.0 k_B_T/e, isovalue 4.0 E^−4^).

All the residues observed at the second RVD position (X13) share with H and N (at X12) a very low intrinsic α-helix propensity [43−45], which is consistent with the observed coil arrangement. As evident from X-ray structures, the side-chain of X13 is closer to the DNA base than X12, thus making X13 the key player in molecular recognition and base discrimination. MD simulations revealed a very low structural fluctuation of the RVD loop (Figure 3A) and energetic analysis showed TAL-DNA binding (Figure 3B/C) dominated by an *oxyanion clip* constituted by G14, K16 and Q17 (GGKQ in Figure 4A). These data taken together suggest a very important role for the *oxyanion clip* in anchoring and spatially constraining the RVD loop, effectively giving very little leeway to the side-chain of X13. Additionally, bulky residues are not compatible at the X13 position because their side-chain would difficultly be accommodated in the narrow gap at TAL-DNA interface.

### A structural model to interpret TAL specificity

The geometrical constraints on the backbone of the RVD loop and the restrictions on both size and conformational freedom of the X13 side-chain are the key elements to understand nucleobase discrimination and amino acid occurrences in RVDs. A simple unified pharmacophore-like model for both purines and pyrimidines has been constructed to highlight the nature and extent of the *dynamical* interactions between the nucleobase and the X13 side-chain over the course of our simulations (Figures 4A). Since only a small portion of the nucleobase is accessible by X13 (loci α, β, γ; corresponding to substituents on C4, C5, C6 for pyrimidines, and C6, N7, C8 for purines), full discrimination between different bases is, in some cases, imperfect and molecular recognition ambiguous, consistent with experimental findings about the shared (none-univocal) specificities of some RVDs (*e.g.* NN, N*, NS) [4], [5], [22].

The pharmacophore-like model, together with the cartoon of Figure 4A, allows to rationalize the RVD-to-DNA code. As an example, N13 (*e.g.* in the NN RVD) clearly favours purine bases over pyrimidine bases owing to the lack of steric clashes with groups on α and β (missing in purines, Figures 4) and to the possibility to form a H-bond with the pyridine-like nitrogen N7 (γ). Nevertheless, recognition based on H-bonding fails to discriminate between adenine and guanine due the impossibility to access – during dynamics – all the loci required for a complete molecular differentiation. This explains the observed selectivity of N13 for both purine bases. NN-containing TALs were recently reported to display greater affinity when targeting guanosine over adenine [22], [41], which is easily understandable by comparing the molecular electrostatic maps of guanine and adenine. Both possess a pyridine-like nitrogen with a lone-pair, which has been postulated to be the interaction centre recognized by asparagine. However, the nearby carbonyl moiety of guanine make the N7 position electrostatically more negative, increasing the strength of interaction with H-bond donors, which is reflected in the respective binding activities. On the contrary, I13 (in NI RVD) perfectly targets adenine on the basis of charge distribution, dipole moment and steric discrimination. Indeed, the larger dipole moment of guanine [46] provides a poorer match for the hydrophobic side-chain of isoleucine and the α/β loci of thymine and cytosine would sterically clash.

Negative discrimination provides a rationale for D13 (in HD and ND RVDs) exclusively targeting cytosine; the carboxylate moiety on D13 forms a stabilizing interaction with the amino group on C4 (β) of cytosine, but would clash with the methyl group on C5 (α) of thymine, and would provide destabilizing interactions with the lone pair of the pyridine-like nitrogen N7 (γ) of either purines. Another illustration of negative discrimination is thymine targeted by NG, HG or N* RVDs. Here, any side-chain would clash with the methyl group at the α locus and thus only absence of side-chain (*i.e.* G or *) can avoid this unfavourable interaction. Finally, even though scarcely present in the crystal structures, the specificity of the NS RVD can also be rationalized with the aid of the pharmacophore-like model and shown to be consistent with experimental findings. Indeed, as for the other amino acids present at position X13, the side-chain of serine is too small to allow a complete sampling of all loci and is therefore only able to properly discriminate thymine on a steric basis, thus agreeing with early observations about serine specificity [4]. More recently [22], NS has been shown to possess a stronger preference for purines over cytosine. Negative discrimination again explains this observation since their respective electrostatic distributions make the side-chain of serine interact better with either purine than with cytosine (Figure 4B).

Based on the interpretation provided by the pharmacophore-like model, the large dominance of non-specific energetic contributions to TAL-DNA binding and the low fluctuation of the RVD loop – resulting in incomplete molecular recognition – we suggest that sequence specificity is not achieved through positive recognition of nucleobases but instead stems from negative discrimination, *i.e.* the match between base and RVD corresponds to the least bad option available when taking steric and electrostatic contributions into account. Furthermore, the little space available at the TAL-DNA interface only allows for small amino acids to be present, thus providing an explanation for the subset composition of the naturally-occurring RVDs. Energy calculations showed that RVD-DNA interactions account for only a small portion of the total binding energy (Figure 3B/C), nonetheless, the fact that multiple repeats (at least 10.5) are needed to produce full activity [5] suggests that the free energy contributions (*i.e.* enthalpy and entropy) of each repeat to TAL binding are likely on a similar scale; any small RVD mismatch would have an energetic cost that would turn protein-DNA binding into an unfavourable process (Figure S12).

The pharmacophore-like model can also be used to predict DNA-recognition by uncommon RVDs that are not present in the crystal structures available. Targeting guanosine is a concern in biotechnological applications since NN is the only common RVD recognizing this base but displays selectivity for both purines. This prompted the search for an alternative. Cong *et al.* recently reported the rare NH RVD as displaying high specificity for guanine while retaining biological activity when incorporated into TAL constructs [47]. Application of the pharmacophore-like model explains this selectivity; the bulky imidazole ring of histidine would clash with both cytosine and thymine (loci α, β) making purine bases a less bad option. The high discrimination between adenine and guanine could be attributed to the p*Ka* of histidine, making the charged histidine a much better match to the electrostatic potential displayed by guanine as compared with that of adenine (Figure 4B). Those findings were further supported by Streubel *et al.*, who demonstrated that NH was selective for guanine although reducing activity of TAL constructs [22]. The size of the imidazole ring and the resulting steric crowding is likely to be the source of the reduced efficiency associated with this RVD.

Lysine (in NK RVD) has even greater specificity for guanine [6], [7], [22], [48]; the positively charged amino moiety at the tip of the long side-chain interacts better with guanine than with adenine or cytosine, due to differences in charge distribution (*cf.* electron densities at loci β, γ and ε in Figure 4B). On the other side, the methyl group on cytosine C5 would generate destabilizing steric clashes with the long lysine side-chain, providing another example of negative discrimination.

### TAL design and DNA methylation

**Figure 5:**
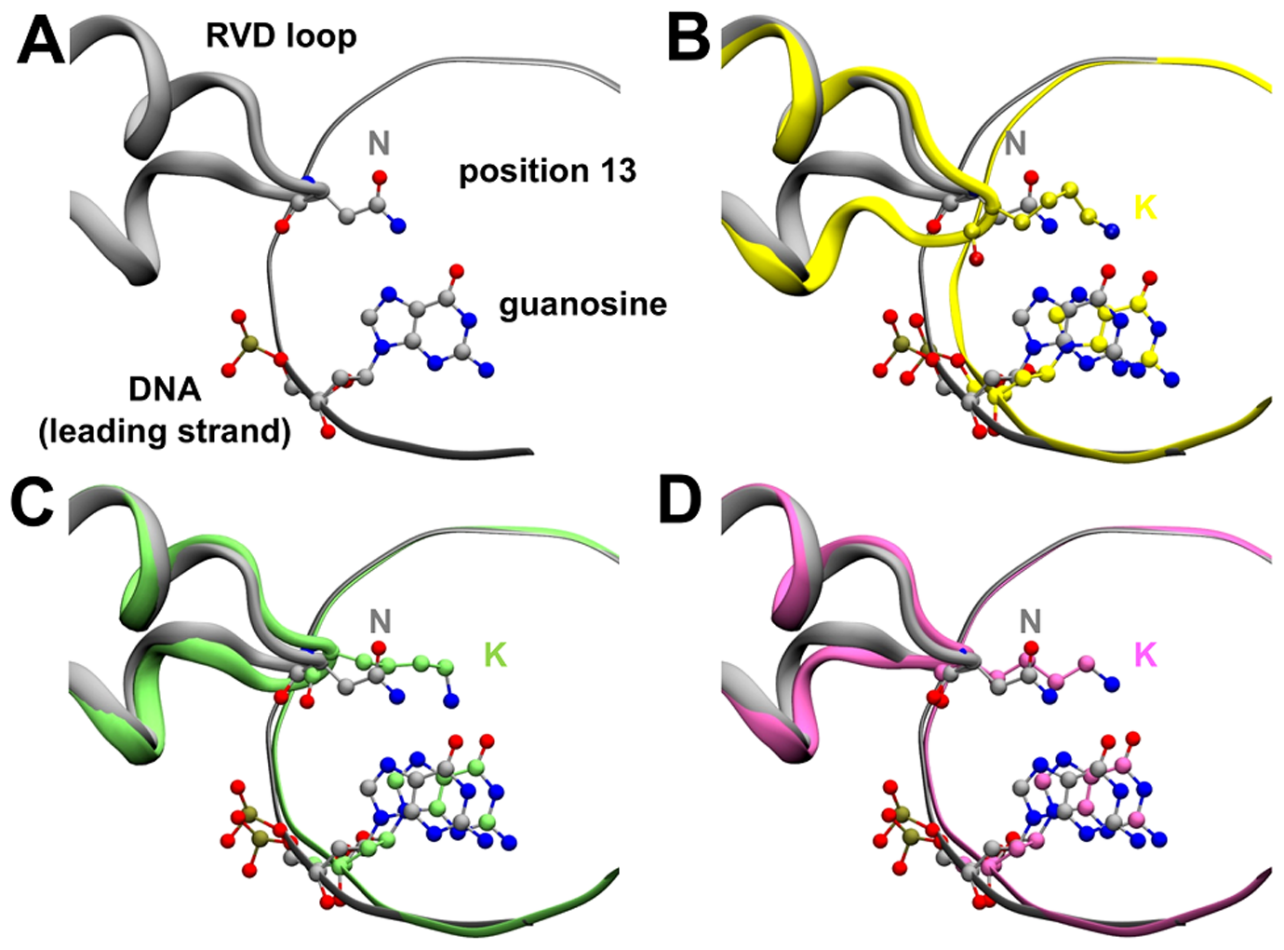
Protein-DNA interactions of potentially improved RVD loops for targeting guanine. (**A**) Wild type (N12-N13) alone (grey) and superposed to: (**B**) N12-N13K mutant (yellow), (**C**) N13K-G14* mutant (green) and (**D**) N13*-G14K mutant (magenta).

Although NK binds guanine more specifically than NN does, it leads to less efficient TAL constructs [22], [49]. The lysine side-chain, due to its size, probably does not perfectly fit in the narrow gap between the RVD loop and DNA, thus impairing binding. We used geometry optimization of single and double TAL mutants to generate new RVD loops *in silico,* which we hope will retain the stronger selectivity of K13 for guanine without overall loss in binding efficiency (*cf.* Figure 5 and Methods S1). We supposed that deletions in the RVD loop could provide the extra space needed to properly accommodate the large side-chain of lysine and not interfere with the DNA-binding. On the basis of our calculations, we propose that the mutants *13-G14K (deletion of X13 and mutation G14K) and K13-*14 (deletion of G14) could be used as effective guanine-targeting RVDs, replacing both NK and NN.

Finally, extension of the pharmacophore-like model to methylated DNA bases helps understanding the shared affinity of the N* RVD for thymine and cytosine as well as providing insight into DNA methylation with respect to TAL binding [4], [5] (Methods S1). DNA methylation, in particular the formation of 5-methylcytosine (mC) is frequent in plants [50] and is also an important epigenetic regulatory mechanism. Modelling of 5-methylcytosine revealed that locus α features the same steric hindrance and electrostatic distribution as thymine (Figure 4B), thus altering the major-groove facet of cytosine upon methylation. N* lacking a side-chain – preventing discrimination further than the α locus – fails to discriminate between 5-methylcytosine and thymine (and cytosine, which lacks the α locus). Consistent with our model, binding energies are indeed not affected significantly when substituting cytosine for 5-methylcytosine or thymine (Table 2). Those data are in agreement with the similar affinities of N* for cytosine and thymine reported by Moscou *et al.* [4]. It is tempting to speculate that N* might have evolved as a versatile RVD capable of leaving TAL DNA-binding unaffected in case of cytosine C5-methylation. The recent study of Deng *et al.* [23] on 5-methyltcytosine DNA targeted by NG revealed no differences in binding when compared to thymine equivalents. Their crystal structure showed a van der Waals contact between methylated DNA bases and NG that resembles thymine targeting by the same RVD. The lack of side-chain in glycine makes it almost identical to N*; on the basis of the pharmacophore-like model, NG and N* are equivalently treated with respect to nucleobase sampling (Figure 4A). Thus, we suppose that under similar experimental conditions N* would show comparable results as NG.

**Table 2:** Protein-DNA binding energies for modified targets of repeat 7 (N*) of the PthXo1 system (TAL[22.5]/P1).

	MM/GBSA [kcal/mol]	MM/PBSA [kcal/mol]
**Cytosine (wild type)**	−147.36 (+/− 15.96)	−5.87 (+/− 27.74)
**Thymine (with Adenine)**	−148.22 (+/− 15.95)	−7.37 (+/− 27.48)
**5**−**methylcytosine**	−149.77 (+/− 15.90)	−8.21 (+/− 27.35)

The base pairs corresponding to each category are: wild type (CG), mutated (TA) and 5-methylcytosine (mCG). See Methods S1 for details.

## Conclusions

The dynamical study described in this report suggests that the composition of each RVD can be deconvoluted into its constituent parts and their contributions treated separately. X12 only interacts with the protein at the helix-RVD-loop transition. Together with the low α-helix propensities of both H and N, it suggests a purely structural role for X12, associated with the disruption of the α1 helix in order to allow proper folding of the RVD-loop and dense wrapping of DNA. By extension, X13 seems to be the sole player in DNA sequence recognition. However, the energetic pattern of TAL-DNA binding shows a strong dominance of the *oxyanion clip* (GGKQ) and a small contribution from X13. This suggests a mechanism of negative discrimination between X13 and the nucleobase. The low structural fluctuation of the RVD-loop imposed by the *oxyanion clip* in the bound state allowed us to devise a pharmacophore-like model for rationalization of the RVD-to-DNA code. The structurally-imposed incomplete molecular recognition of nucleobases by X13 explains the selectivity of the different RVDs and their shared affinities. The observation of a significant interaction between X13 and the preceding DNA base might provide an explication for the context-dependence of TAL activities. We hope that the recognition mechanism proposed herein will allow for more efficient rational designs, deepening alongside our knowledge of the intricate, yet very elegant architecture of TAL proteins.

## Supporting Information

Figure S1 **Root Mean Square Deviations (RMSD)**. Calculations performed on C_α_ and P atoms for different portions of the systems (labels in the upper-right box of each graph). The overall RMSD for each portion as well as the corresponding standard deviation (in brackets) are reported next to each label. (**a**) TAL[22.5]/P1, (**b**) TAL[22.5]/P2, (**c**) TAL[11.5]/P1, (**d**) TAL[11.5]/P1-apo, (**e**) TAL[11.5]/P3, (**f**) TAL[11.5]/P4 and (**g**) TAL[10]/P1-apo. Analyses done with ProDy and plotted with Matplotlib Python libraries.
(TIF)

Figure S2 **Projection of the first four normal modes onto the trajectory**. Normal modes obtained by Essential Dynamics Analysis (protein C_α_ atoms) of the MD trajectories. Labels are reported in the upper-right box of each graph, together with the statistical weight of each normal mode (in brackets). (**a**) TAL[22.5]/P1, (**b**) TAL[22.5]/P2, (**c**) TAL[11.5]/P1, (**d**) TAL[11.5]/P1-apo, (**e**) TAL[11.5]/P3, (**f**) TAL[11.5]/P4 and (**g**)TAL[10]/P1-apo. Analyses were done with ProDy and plotting with Matplotlib Python libraries.
(TIF)

Figure S3 **Root Mean Square Fluctuation (RMSF)**. Calculations performed on protein C_α_ atoms. (**a**) TAL[22.5]/P1, (**b**) TAL[22.5]/P2, (**c**) TAL[11.5]/P1, (**d**) TAL[11.5]/P1-apo, (**e**) TAL[11.5]/P3, (**f**) TAL[11.5]/P4 and (**g**)TAL[10]/P1-apo. Analyses done with ProDy and plotting with Matplotlib Python libraries.
(TIF)

Figure S4 **Secondary structure time evolution**. The colours represent the different secondary structure elements (blue: α-helix; white: coil/turn; orange: β-sheet). (**a**) TAL[22.5]/P1, (**b**) TAL[22.5]/P2, (**c**) TAL[11.5]/P1, (**d**) TAL[11.5]/P1-apo, (**e**) TAL[11.5]/P3, (**f**) TAL[11.5]/P4 and (**g**)TAL[10]/P1-apo. Analyses done with VMD and plotting with Matplotlib Python libraries.
(TIF)

Figure S5 **Average Root Mean Square Fluctuation (RMSF) per TAL repeat**. Calculations performed on protein C_α_ atoms. (**a**) TAL[22.5]/P1, (**b**) TAL[22.5]/P2, (**c**) TAL[11.5]/P1, (**d**) TAL[11.5]/P1-apo, (**e**) TAL[11.5]/P3, (**f**) TAL[11.5]/P4 and (**g**)TAL[10]/P1-apo. Repeats containing a deletion were excluded from the statistics. Standard deviation values are reported as error bars. Analyses done with ProDy and plotting with Matplotlib Python libraries.
(TIF)

Figure S6 **Per-residue decomposition of the intra-protein total interaction energy**. Calculations performed on the TAL[22.5]/P1 system using the MM/GBSA (single-trajectory) approach. Graph obtained by taking the average per repeat and displaying a three-repeat window. For clarity, only values below −0.5 kcal/mol are reported.
(TIF)

Figure S7 **Per-residue decomposition of the intra-protein interaction energy (only backbone contributions)**. Calculations performed on the TAL[22.5]/P1 system using the MM/GBSA (single-trajectory) approach. Graph obtained by taking the average per repeat and displaying a three-repeat window. For clarity, only values below −0.5 kcal/mol are reported.
(TIF)

Figure S8 **Per-residue decomposition of the intra-protein interaction energy (only side-chain contributions)**. Calculations performed on the TAL[22.5]/P1 system using the MM/GBSA (single-trajectory) approach. Graph obtained by taking the average per repeat and displaying a three-repeat window. For clarity, only values below −0.5 kcal/mol are reported.
(TIF)

Figure S9 **Decomposition of the protein-DNA total interaction energy of TAL[22.5]/P1**. Calculations performed on the model system TAL[22.5]/P1 using the MM/GBSA (single-trajectory) approach. For clarity, only values below −0.5 kcal/mol are reported.
(TIF)

Figure S10 **Decomposition of the protein-DNA total interaction energy of TAL[11.5]/P1**. Calculations performed on the model system TAL[11.5]/P1 using the MM/GBSA (single-trajectory) approach. For clarity, only values below −0.5 kcal/mol are reported.
(TIF)

Figure S11 **Contributions from different sections of TAL to the total protein-DNA binding energy (subdivided by type)**. Calculation performed using the MM/GBSA (single-trajectory) approach. (**a**) TAL[22.5]/P1, (**b**) TAL[22.5]/P2, (**c**) TAL[11.5]/P3, (**d**) TAL[11.5]/P4 and (**e**) TAL[11.5]/P1. Colour-code of the repeat-specific bars; red = G13, yellow = N13, blue = D13 and green = I13 (**a** and **b**) or green = S13 (**c**, **d** and **e**).
(TIF)

Figure S12 **Contributions from an increasing number of TAL repeats to the total protein-DNA binding energy**. Calculations performed using the MM/GBSA and MM/PBSA (single-trajectory) approaches on model system TAL[22.5]/P1 (*cf.* Methods S1 for details).
(TIF)

Methods S1 Supporting methods and references.
(PDF)
