# Supplementary figures and images for: TAL Effectors Specificity Stems from Negative Discrimination
Source: PLoS One. 2013 Nov 25;8(11):e80261. doi: 10.1371/journal.pone.0080261 (PMC3840011; doi:10.1371/journal.pone.0080261)

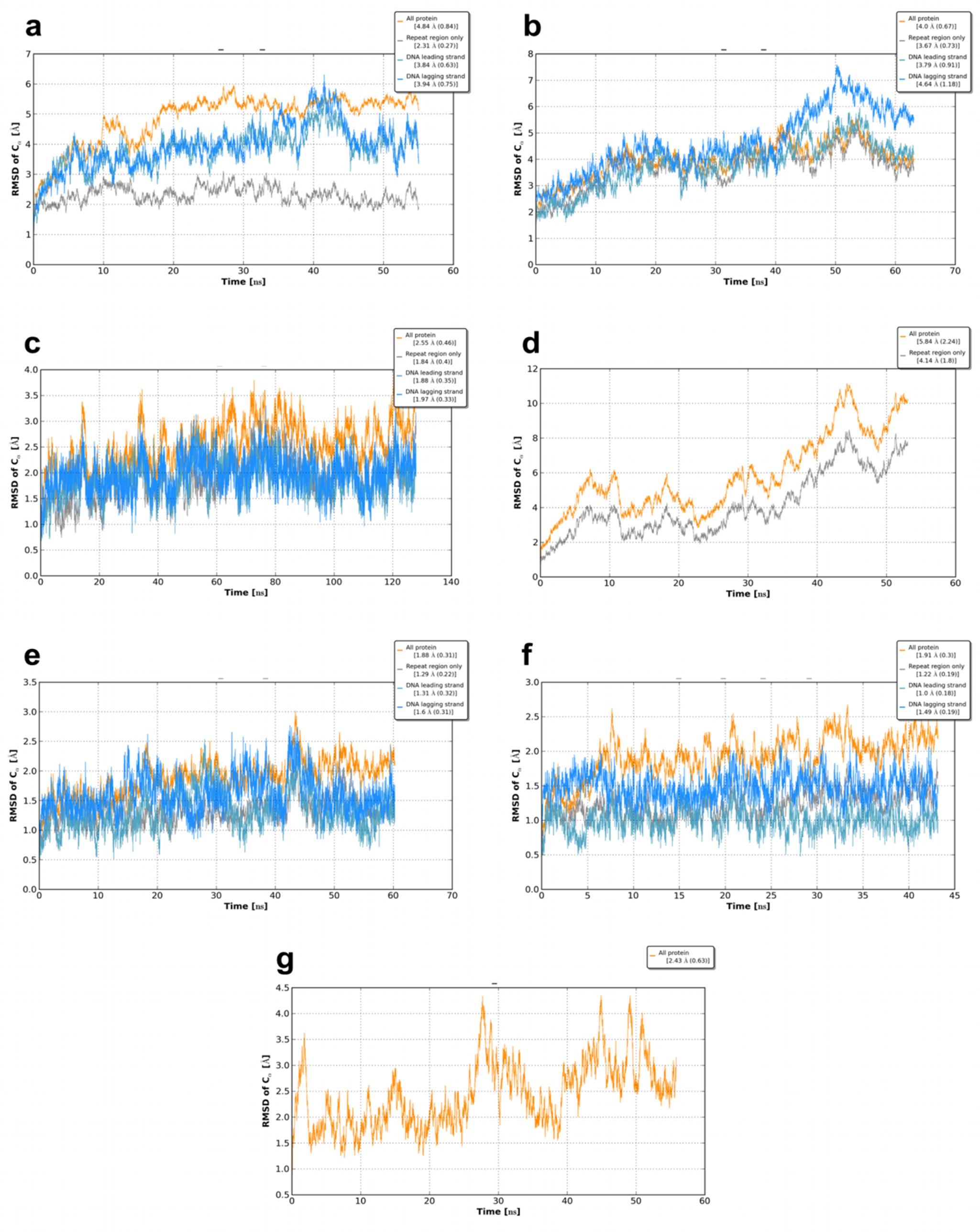

Supplement: Figure S1 — Root Mean Square Deviations (RMSD). Calculations performed on Cα and P atoms for different portions of the systems (labels in the upper-right box of each graph). The overall RMSD for each portion as well as the corresponding standard deviation (in brackets) are reported next to each label. (a) TAL[22.5]/P1, (b) TAL[22.5]/P2, (c) TAL[11.5]/P1, (d) TAL[11.5]/P1-apo, (e) TAL[11.5]/P3, (f) TAL[11.5]/P4 and (g) TAL[10]/P1-apo. Analyses done with ProDy and plotted with Matplotlib Python libraries. (TIF) [file pone.0080261.s001.tif]

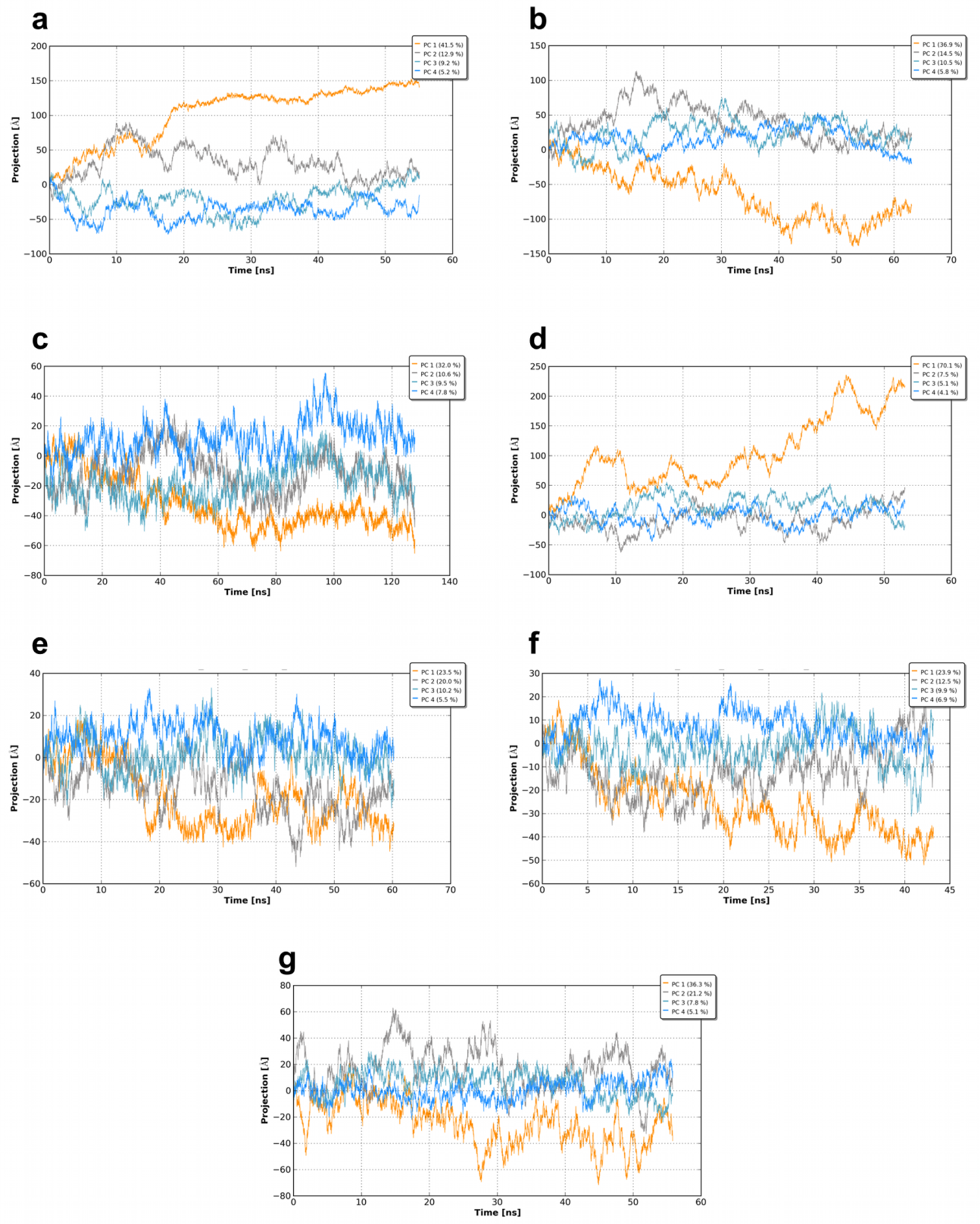

Supplement: Figure S2 — Projection of the first four normal modes onto the trajectory. Normal modes obtained by Essential Dynamics Analysis (protein Cα atoms) of the MD trajectories. Labels are reported in the upper-right box of each graph, together with the statistical weight of each normal mode (in brackets). (a) TAL[22.5]/P1, (b) TAL[22.5]/P2, (c) TAL[11.5]/P1, (d) TAL[11.5]/P1-apo, (e) TAL[11.5]/P3, (f) TAL[11.5]/P4 and (g)TAL[10]/P1-apo. Analyses were done with ProDy and plotting with Matplotlib Python libraries. (TIF) [file pone.0080261.s002.tif]

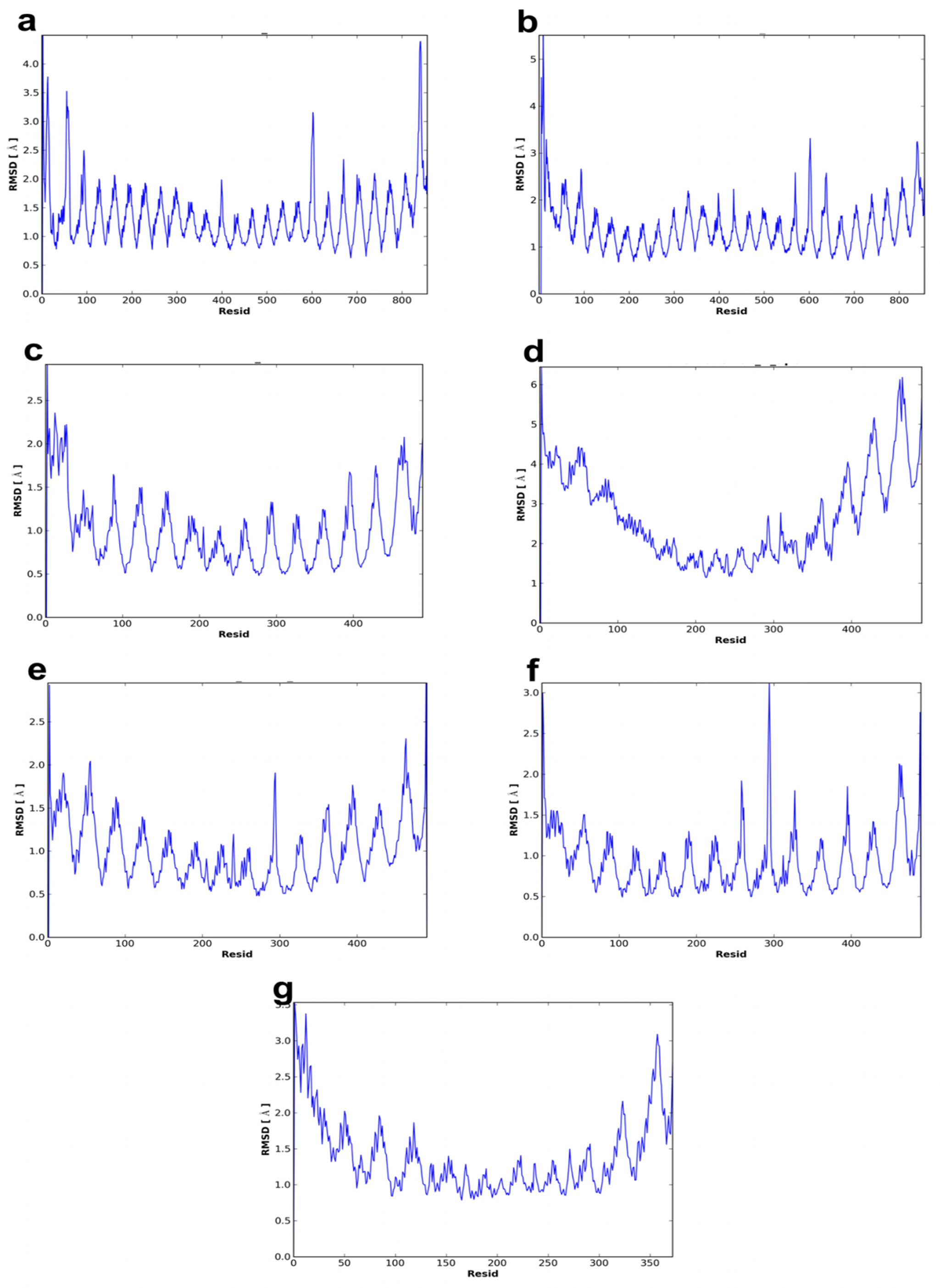

Supplement: Figure S3 — Root Mean Square Fluctuation (RMSF). Calculations performed on protein Cα atoms. (a) TAL[22.5]/P1, (b) TAL[22.5]/P2, (c) TAL[11.5]/P1, (d) TAL[11.5]/P1-apo, (e) TAL[11.5]/P3, (f) TAL[11.5]/P4 and (g)TAL[10]/P1-apo. Analyses done with ProDy and plotting with Matplotlib Python libraries. (TIF) [file pone.0080261.s003.tif]

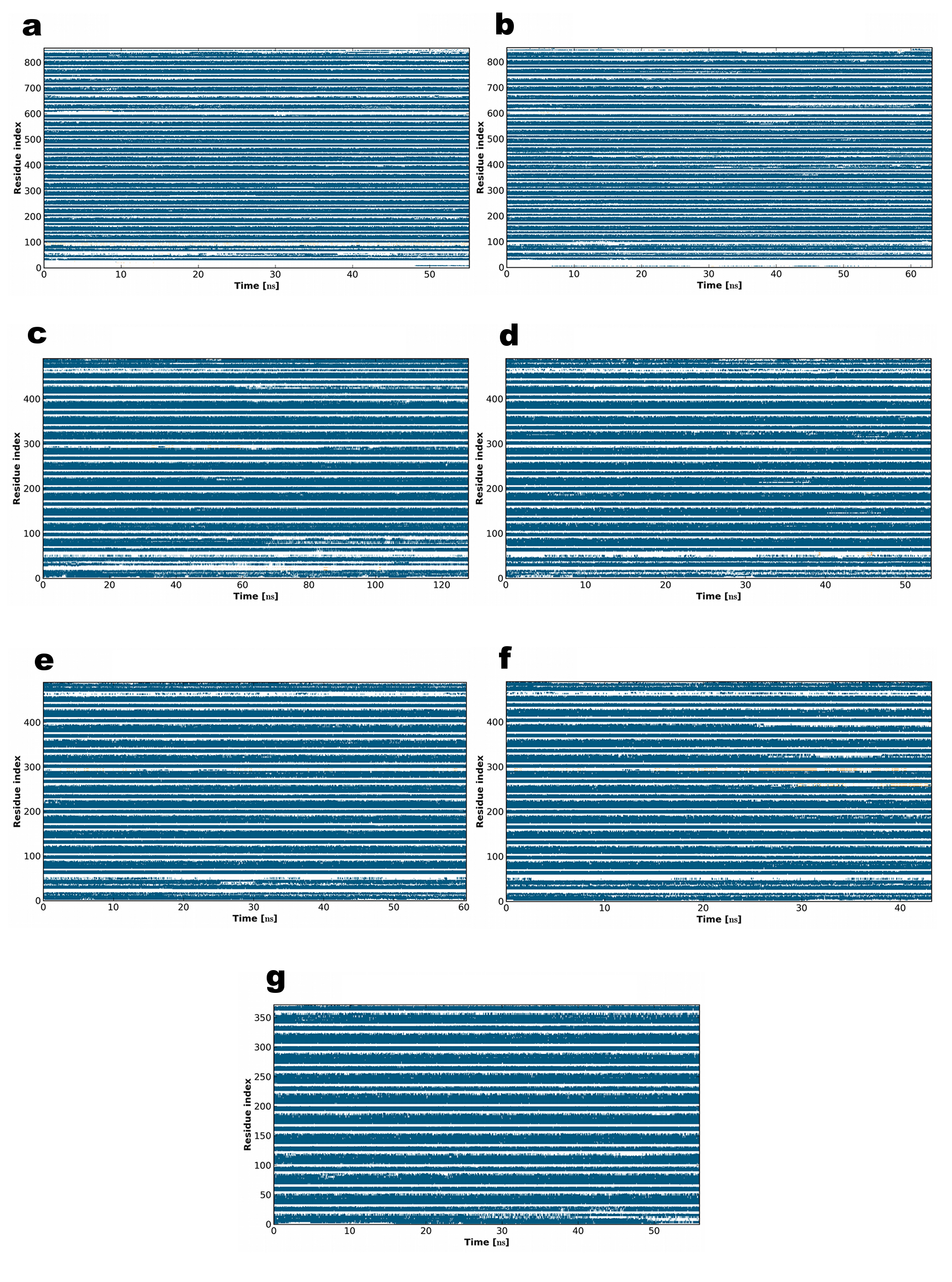

Supplement: Figure S4 — Secondary structure time evolution. The colours represent the different secondary structure elements (blue: α-helix; white: coil/turn; orange: β-sheet). (a) TAL[22.5]/P1, (b) TAL[22.5]/P2, (c) TAL[11.5]/P1, (d) TAL[11.5]/P1-apo, (e) TAL[11.5]/P3, (f) TAL[11.5]/P4 and (g)TAL[10]/P1-apo. Analyses done with VMD and plotting with Matplotlib Python libraries. (TIF) [file pone.0080261.s004.tif]

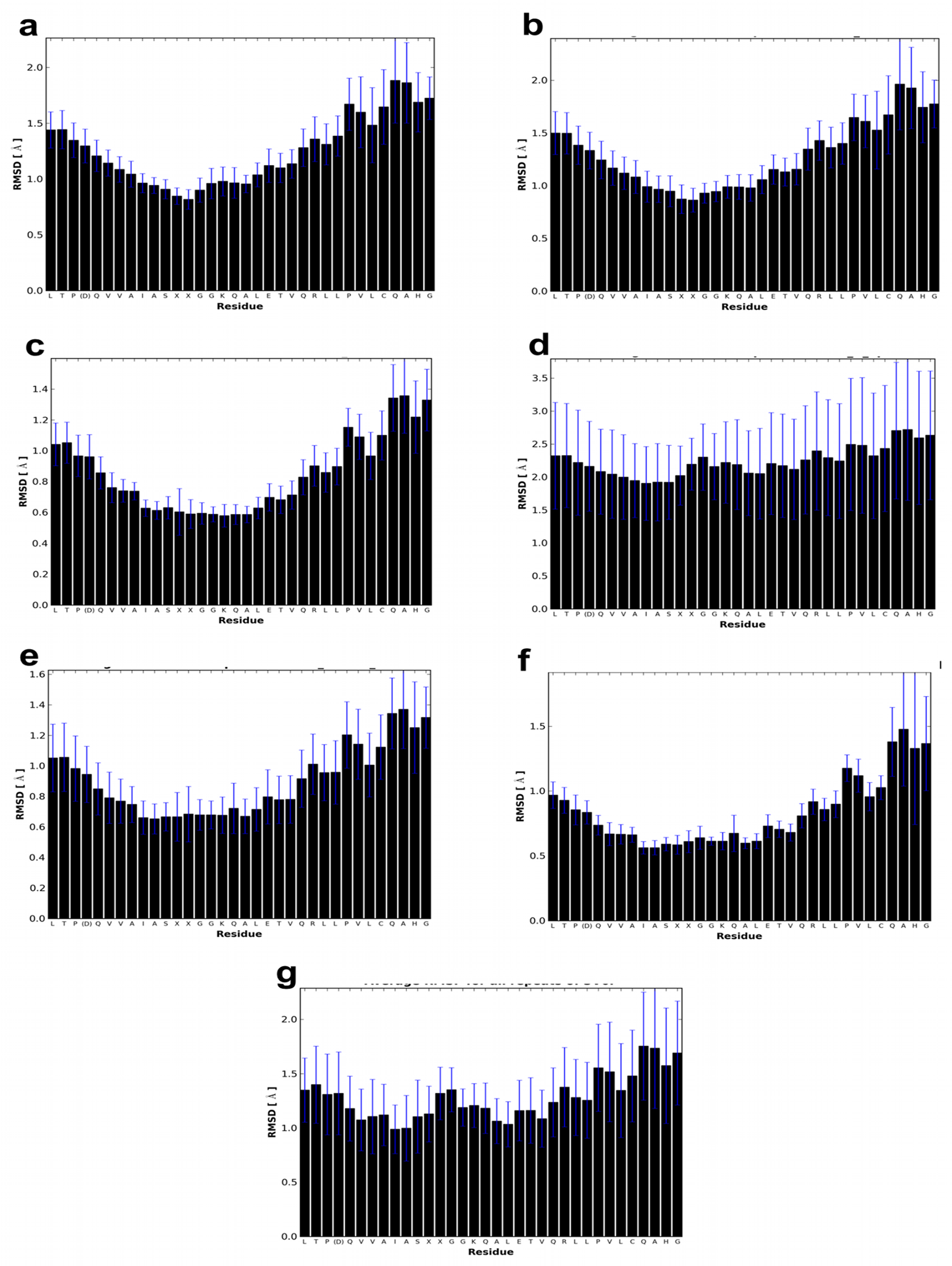

Supplement: Figure S5 — Average Root Mean Square Fluctuation (RMSF) per TAL repeat. Calculations performed on protein Cα atoms. (a) TAL[22.5]/P1, (b) TAL[22.5]/P2, (c) TAL[11.5]/P1, (d) TAL[11.5]/P1-apo, (e) TAL[11.5]/P3, (f) TAL[11.5]/P4 and (g)TAL[10]/P1-apo. Repeats containing a deletion were excluded from the statistics. Standard deviation values are reported as error bars. Analyses done with ProDy and plotting with Matplotlib Python libraries. (TIF) [file pone.0080261.s005.tif]

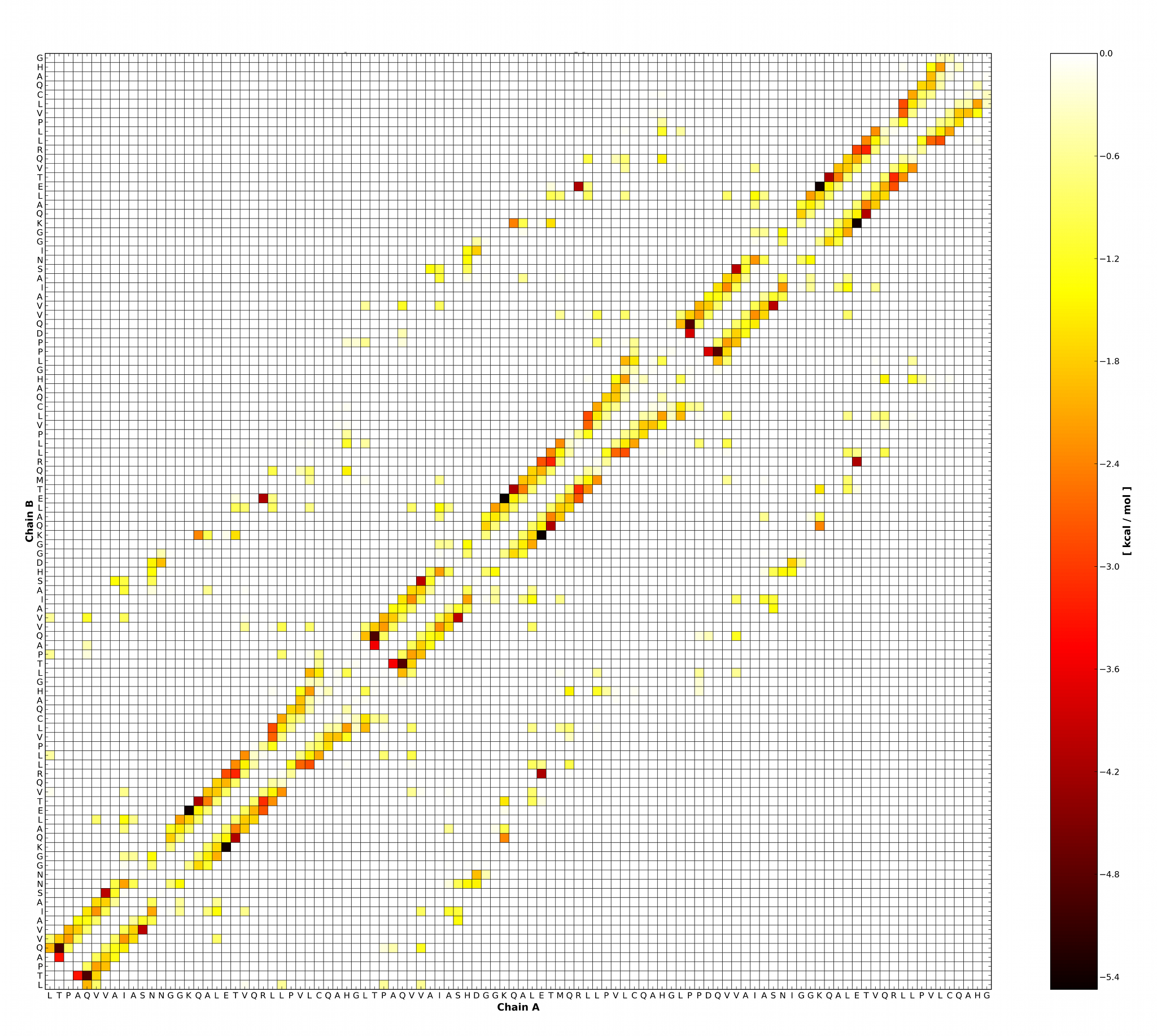

Supplement: Figure S6 — Per-residue decomposition of the intra-protein total interaction energy. Calculations performed on the TAL[22.5]/P1 system using the MM/GBSA (single-trajectory) approach. Graph obtained by taking the average per repeat and displaying a three-repeat window. For clarity, only values below −0.5 kcal/mol are reported. (TIF) [file pone.0080261.s006.tif]

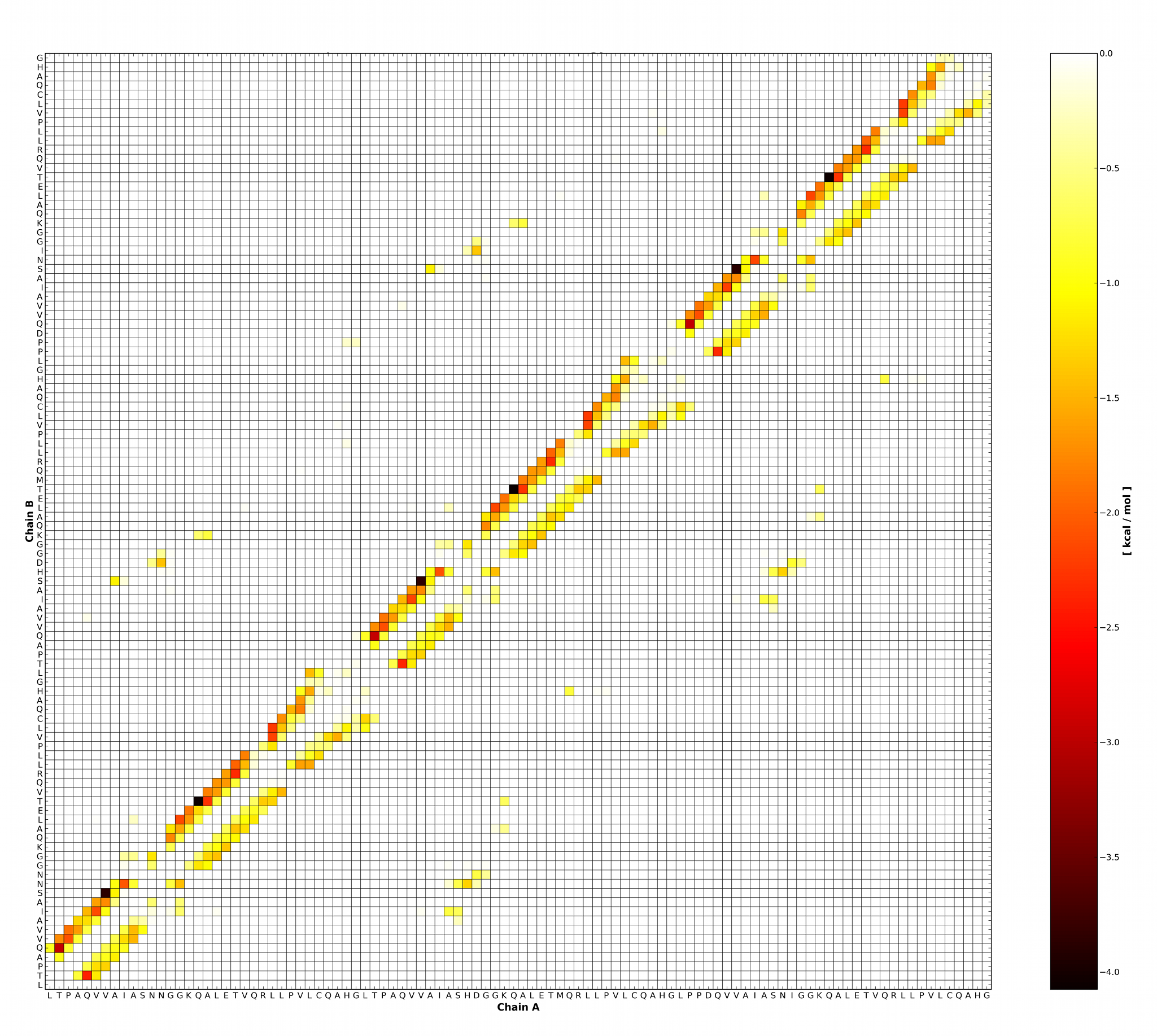

Supplement: Figure S7 — Per-residue decomposition of the intra-protein interaction energy (only backbone contributions). Calculations performed on the TAL[22.5]/P1 system using the MM/GBSA (single-trajectory) approach. Graph obtained by taking the average per repeat and displaying a three-repeat window. For clarity, only values below −0.5 kcal/mol are reported. (TIF) [file pone.0080261.s007.tif]

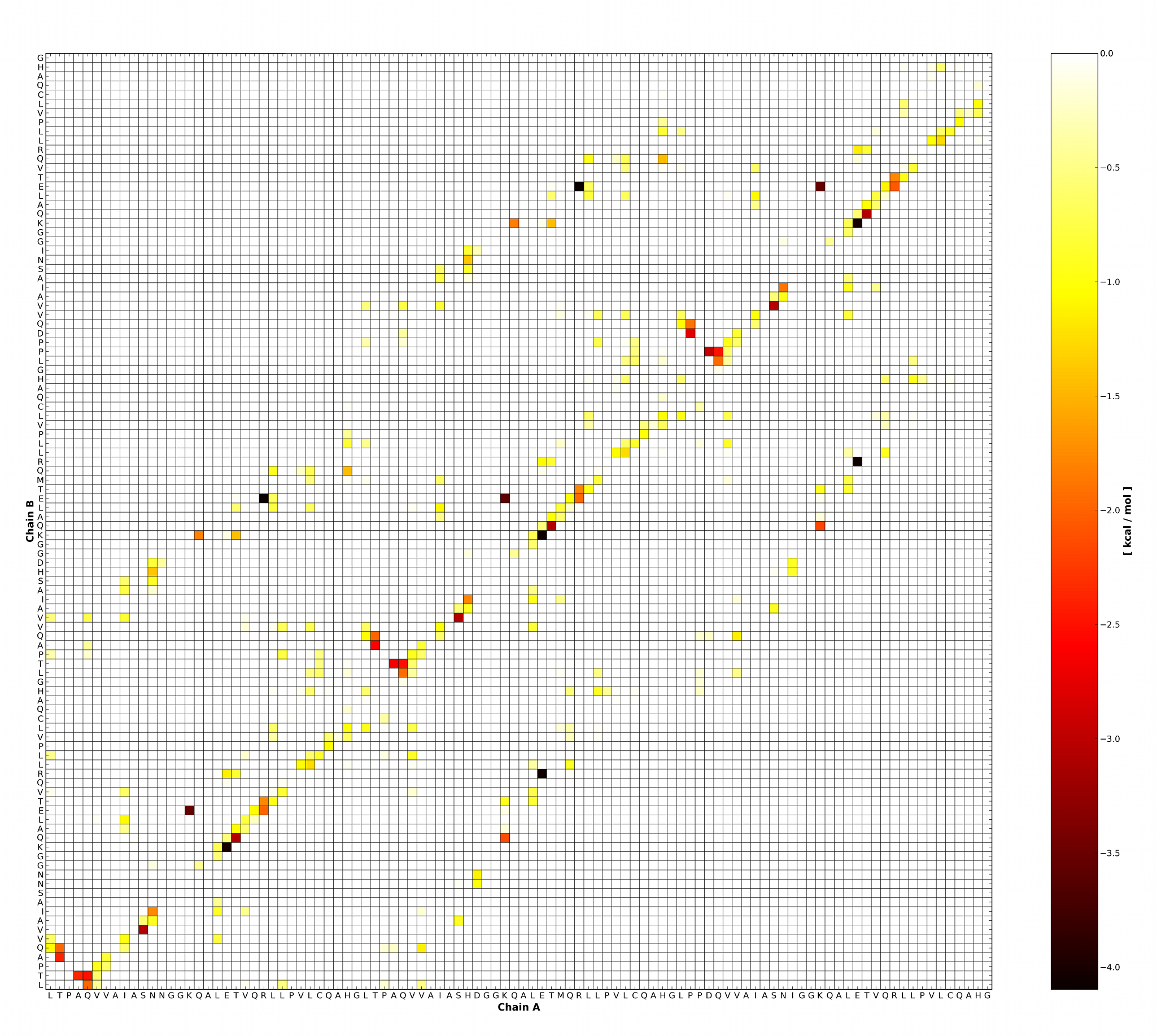

Supplement: Figure S8 — Per-residue decomposition of the intra-protein interaction energy (only side-chain contributions). Calculations performed on the TAL[22.5]/P1 system using the MM/GBSA (single-trajectory) approach. Graph obtained by taking the average per repeat and displaying a three-repeat window. For clarity, only values below −0.5 kcal/mol are reported. (TIF) [file pone.0080261.s008.tif]

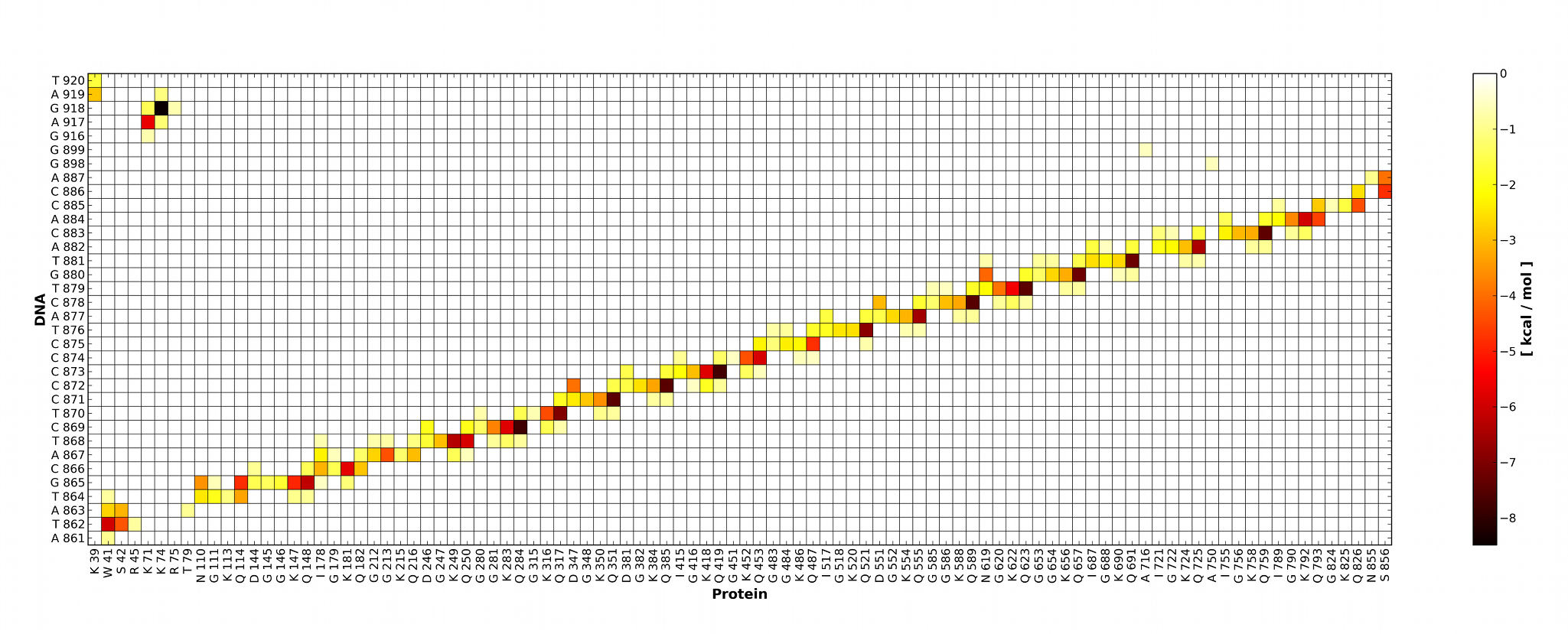

Supplement: Figure S9 — Decomposition of the protein-DNA total interaction energy of TAL[22.5]/P1. Calculations performed on the model system TAL[22.5]/P1 using the MM/GBSA (single-trajectory) approach. For clarity, only values below −0.5 kcal/mol are reported. (TIF) [file pone.0080261.s009.tif]

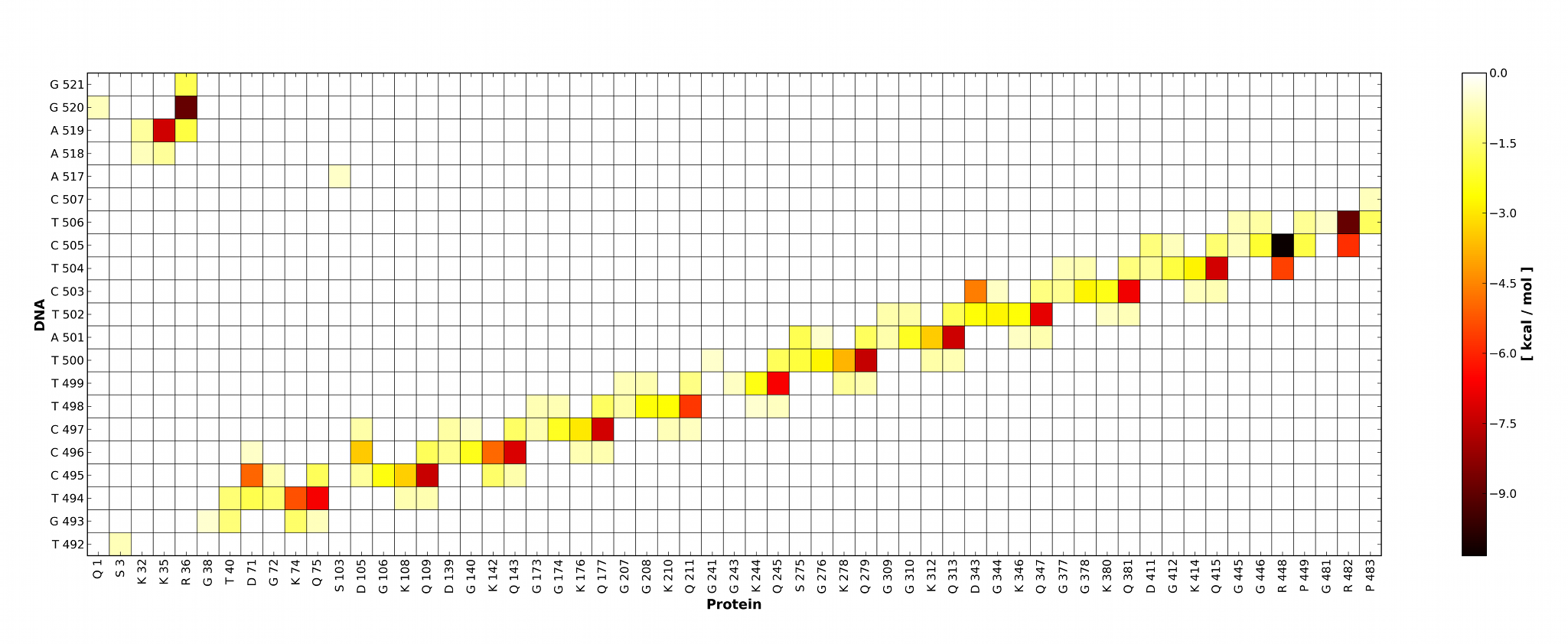

Supplement: Figure S10 — Decomposition of the protein-DNA total interaction energy of TAL[11.5]/P1. Calculations performed on the model system TAL[11.5]/P1 using the MM/GBSA (single-trajectory) approach. For clarity, only values below −0.5 kcal/mol are reported. (TIF) [file pone.0080261.s010.tif]

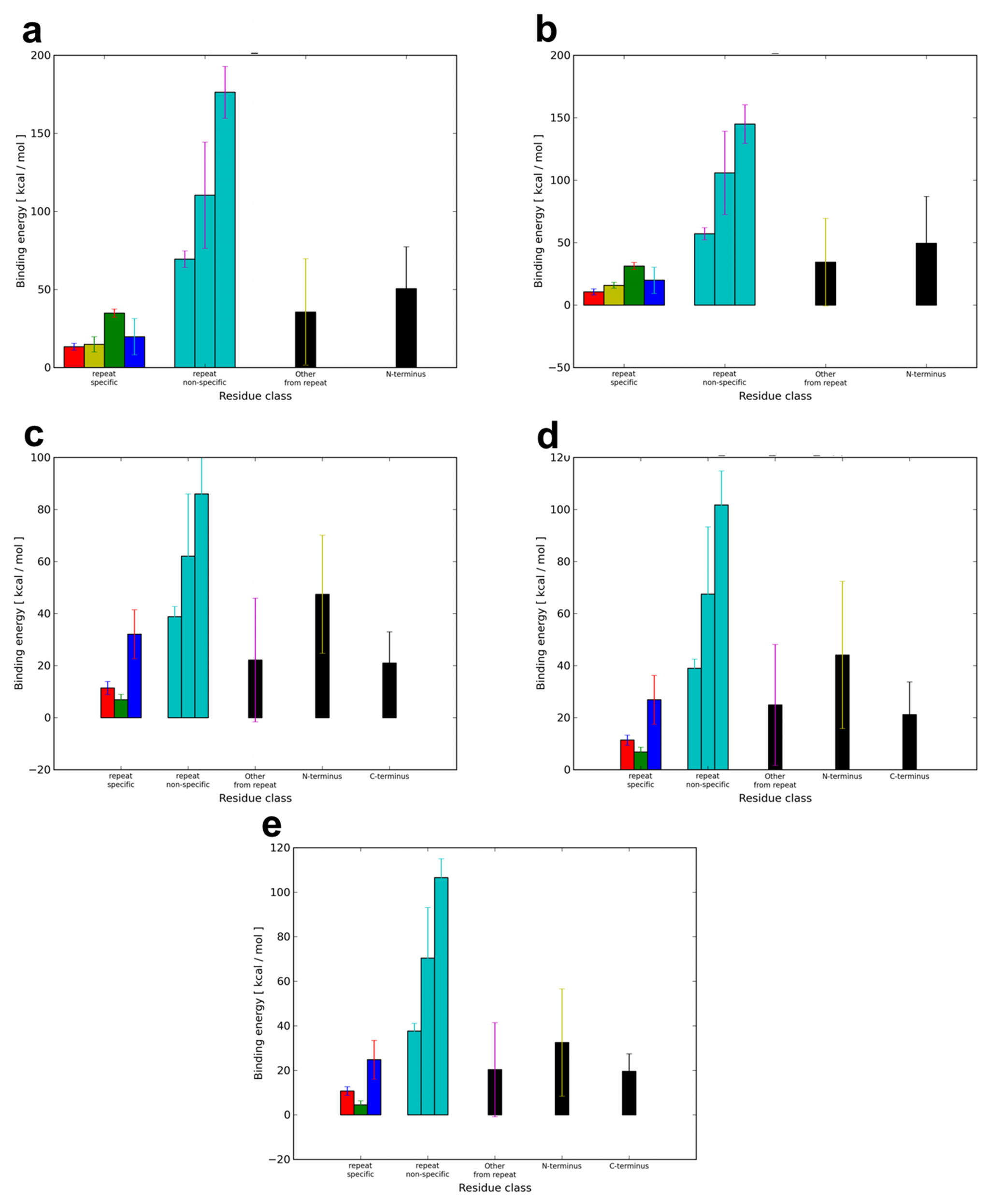

Supplement: Figure S11 — Contributions from different sections of TAL to the total protein-DNA binding energy (subdivided by type). Calculation performed using the MM/GBSA (single-trajectory) approach. (a) TAL[22.5]/P1, (b) TAL[22.5]/P2, (c) TAL[11.5]/P3, (d) TAL[11.5]/P4 and (e) TAL[11.5]/P1. Colour-code of the repeat-specific bars; red = G13, yellow = N13, blue = D13 and green = I13 (a and b) or green = S13 (c, d and e). (TIF) [file pone.0080261.s011.tif]

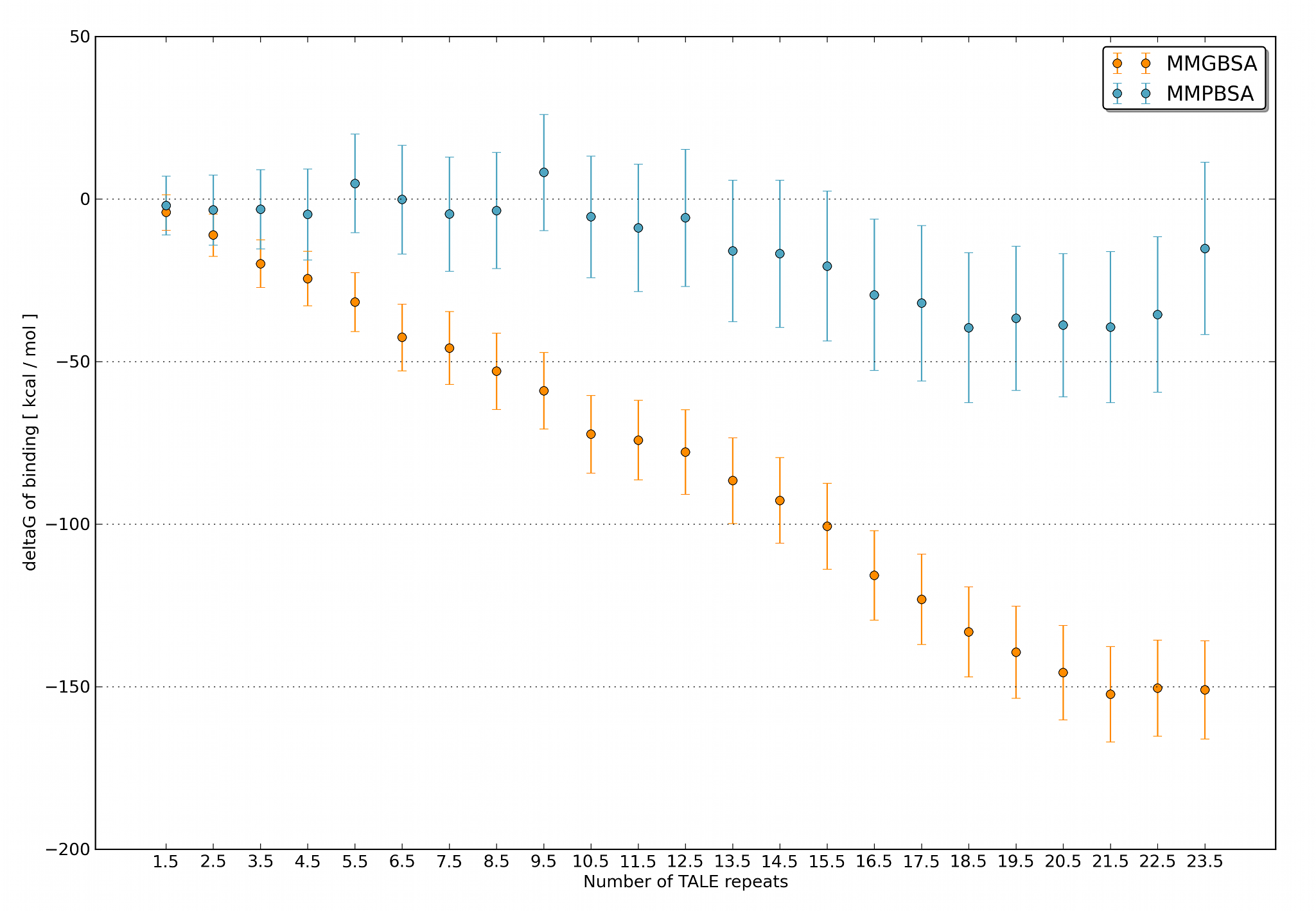

Supplement: Figure S12 — Contributions from an increasing number of TAL repeats to the total protein-DNA binding energy. Calculations performed using the MM/GBSA and MM/PBSA (single-trajectory) approaches on model system TAL[22.5]/P1 (cf. Methods S1 for details). (TIF) [file pone.0080261.s012.tif]
