# Supplementary material for: TAL Effectors Specificity Stems from Negative Discrimination
Source: PLoS One. 2013 Nov 25;8(11):e80261. doi: 10.1371/journal.pone.0080261 (PMC3840011; doi:10.1371/journal.pone.0080261)
Supplement: Methods S1 — Supporting methods and references. (PDF) [file pone.0080261.s013.pdf]

Supporting Information Material for

TAL effectors specificity stems from negative discrimination

Basile I. M. Wicky[1], Marco Stenta[1] and Matteo Dal Peraro[1,2]*

[1] Laboratory for Biomolecular Modeling, Institute of Bioengineering, School of Life Sciences, École Polytechnique Fédérale de Lausanne (EPFL), Lausanne, Switzerland

[2] Swiss Institute of Bioinformatics (SIB), Lausanne, Switzerland

Table of Content

SUPPORTING METHODS 2

Set-up and simulation protocol for molecular dynamics 2

Binding energy calculations 4

Incremental contribution of single TAL repeats to the total binding energy 4

Quantum mechanical calculations 4

Pharmacophore-like model generation 5

Improving guanosine targeting 5

Methylation 5

SUPPORTING REFERENCES 6

* To whom correspondence should be addressed:

Tel: +41 21 693 18 61; Fax: +41 21 693 96 65;

Email: matteo.dalperaro@epfl.ch

SUPPORTING METHODS

Set-up and simulation protocol for molecular dynamics

Unless stated otherwise, all systems were set-up and run according to the following protocol.

Classical Molecular Mechanics (MM) [1] was used to calculate the potential energy of the simulated systems. Geometry optimization (OPT) was used to relax the system while Molecular Dynamics (MD) [2] was employed to investigate time dependent properties. Starting structures were obtained from the Protein Data Bank (PDB) [3]. Homology modeling techniques were used to add missing loops or amino acids. All model systems were constructed using the *tleap* module of the AMBER 11 software package.

A pre-equilibrated orthorhombic box of water molecules was added to each system with a padding of 15 Å. Suitable number of counter-ions (Mg^{2+} and Cl^{-}) were added to get neutral systems and to obtain physiological concentrations of Mg^{2+} (~50 mM).

All OPT and MD simulations were performed using NAMD 2.8 [4] in conjunction with the AMBER ff99brsc [5] force filed and the TIP3P [6] model for water molecules. The van der Waals interaction cutoff distances were set at 12 Å and long-range electrostatic forces were computed using the particle-mesh Ewald summation method with a grid size set to 1.0 Å. No switching function was used for van der Waals interactions and the 1-4 contributions were multiplied by a factor of 0.83 to match the AMBER force field requirements. For all simulations, constant temperature (300 K) was enforced using Langevin dynamics with a damping coefficient of 5 ps^{-1}, constant pressure (1 atm) was enforced through the Nosé-Hoover Langevin piston method with a decay period of 100 fs and a damping time constant of 50 fs. A time step of 2 fs was used throughout. Covalent bonds involving hydrogen atoms were constrained using the RATTLE algorithm.

Simulation protocol included an equilibration phase with all the protein and nucleic acid heavy atoms (*i.e.* not H) constrained by a harmonic potential of force constant 200 kcal/$Å^2$, followed by a production phase with all atoms free.

Equilibration started with 1000 steps of minimization followed by 500 ps of NVT (100 K) simulation for water pre-equilibration. The temperature was gently raised from 100 K to 300 K in 300 ps. A second equilibration phase of 500 ps in the NVT (300 K) ensemble completed the equilibration. The production phase was run in the NPT ensemble (1 atm, 300 K).

Analyses on the trajectories were performed using the ProDy [7] software and the VMD [8] software plug-ins. Pictures were rendered using VMD. Graphs and plots were generated using the Matplotlib [9] Python library. An overview of the simulated systems can found in Table 1 of the main text. The structural analyses are reported in Figure S1-S5.

TAL[22.5]/P1. The initial geometry was taken from the PDB structure 3UGM [10]. Residues 192 to 1048 of chain A were used to model the protein (22.5 repeats instead of the full 23.5 repeats). The few missing residues (5 outer-loops) were re-constructed by homology modeling with the on-line server SWISS-PROT [11]. All DNA residues were kept except residues 30 and 31 for chain B and residues

37 and 38 for chain C, which correspond to the over-hanging 3' and 5' bases of DNA. Crystallization water molecules were maintained. Histidine residues part RVDs as well as His 199/212/276/864/932/966 (part of outer-loops) were protonated on the Nδ atom in order to maximize H-bonding. The other histidines were protonated on the Nε atom. The final system included 77 Mg^{2+} ions and 80 Cl^{-}. System set-up and simulation were performed according to the general procedure with the exception of the harmonic constraint that was set to 100 kcal/A^2 during the equilibration phase. After equilibration, production phase was performed for 53 ns (Number of atoms: 158'675; Box size: 106 × 151 × 107 $Å^3$).

<u>TAL[22.5]/P2</u>. System set-up was performed according to the protocol described above for TAL[22.5]/P1. Histidine residues were protonated as in TAL[22.5]/P1 except for those part of RVDs, which were protonated on the Nε atom. 77 Mg^{2+} and 80 Cl^{-} ions were added to the system. Set-up and simulation were done according to the general simulation procedure except for the equilibration phase. Production phase lasted 61 ns (Number of atoms: 158'675; Box size: 106 × 151 × 107 $Å^3$).

<u>TAL[11.5]/P1</u>. The initial geometry was taken from the PDB structure 3V6T [12]. Chains A (protein), I and J (DNA) were used to create the model system without deleting or adding any residue. Crystallization water molecules were maintained. Histidine residues were protonated on the Nε atom except for those in the RVDs, which were protonated on the Nδ atom. 42 Mg^{2+} and 55 Cl^{-} were added to the system. After standard equilibration, production was performed for 127 ns (Number of atoms: 107'599; Box size: 101 × 102 × 112 $Å^3$).

<u>TAL[11.5]/P3</u>. System set-up was performed according to the protocol described above for TAL[11.5]/P1. Histidine residues were protonated on the Nε atom except for those in RVDs, which were protonated on both the Nδ and Nε atoms (positively charged imidazole ring). 38 Mg^{2+} and 52 Cl^{-} ions were added to the system. After standard equilibration, production was performed for 57 ns (Number of atoms: 107'618; Box size: 101 × 102 × 112 $Å^3$).

<u>TAL[11.5]/P4</u>. System set-up was performed according to the protocol described above for TAL[11.5]/P3. Additionally, imidazole ring atoms were flipped compared to their crystal structure assignments. 38 Mg^{2+} and 52 Cl^{-} ions were added to the system. After standard equilibration, production was performed for 42 ns. (Number of atoms: 107'618; Box size: 101 × 102 × 112 $Å^3$).

<u>TAL[11.5]/P1-apo</u>. The initial geometry was taken from the PDB structure 3V6T [12]. Only chain A (*i.e.* only protein, no DNA) was used to create the model system, without deleting or adding any residue of that chain. Crystallization water molecules were maintained. Histidine residues were protonated on the Nε atom except for those in RVDs, which were protonated on the Nδ atom, thus resulting in a system identical to TAL[11.5]/P1, except for the absence of the double-stranded DNA. 27 Mg^{2+} and 57 Cl^{-} were added to the system. After standard equilibration, production was performed for 51 ns (Number of atoms: 107'635; Box size: 101 × 102 × 112 $Å^3$).

<u>TAL[10]/P1-apo</u>. The initial geometry was taken from the PDB structure 3V6P [12]. This crystal structure does not contain DNA. Histidine residues were protonated on the Nε atom except for those in RVDs, which were protonated on the Nδ atom. Crystallization water molecules were maintained. The protein sequence is identical to that of TAL[11.5], but a few residues at both the N- and C-termini were

not resolved in the crystal structure. 24 Mg^{2+} and 51 Cl^- ions were added to the system. After standard equilibration, production was performed for 56 ns (Number of atoms: 95'742; Box size: 108 × 96 × 99 $Å^3$).

Binding energy calculations

The standard single-trajectory approach was used to calculate binding energy according to either MM/GBSA (Molecular Mechanics/Generalized Born Surface Area) or MM/PBSA (Molecular Mechanics/Poisson Boltzmann Surface Area) methods [13]. Binding free energy decomposition was performed (only MM/GBSA) in order to identify hot spots at the protein-DNA interface [14,15]. All calculations were performed using the parallelized version of MM/PB(GB)SA implemented in the Amber 11 suite of program [16]. An overall salt-concentration of 0.150 M was used for both GB and PB calculations; the program's default parameters were used in either case to calculate the non-polar contributions. In all cases 1-4 terms were included in the internal potential. Entropy contributions were not taken into account. Each MM/PB(GB)SA calculation was performed on 500 snapshots evenly taken from the last 30 ns of equilibrated simulations (one snapshot every 6 ps of MD simulation). The energetic analysis are reported in Figures S6-S11.

Incremental contribution of single TAL repeats to the total binding energy

To understand the dependency between binding energy and the number of repeats wrapping the DNA double-strand, MM/GBSA and MM/PBSA calculations were performed using a non-standard procedure. 500 snapshots from TAL[22.5]/P1 were extracted. Several sets of coordinates were obtained by truncating a number of TAL repeats while keeping intact the DNA double-strand. Each structure set – featuring a different number of TAL repeats but with the same DNA – was re-assembled and subjected to MM/GBSA and MM/PBSA calculations. The results of this analysis are reported in Figure S12. Although the same trend is visible for both implicit solvent models, the quantitative nature of those results should be taken with care. Owing to the nature of the system, in particular the highly charged interaction surface, both PB and GB methods are stressed to their limits [17]. This accounts both for the absolute error of each method and for the discrepancies between the methods, sometimes leading to a worst performance of PB with respect to GB, despite the supposed higher accuracy of the former [18]. It is also worth mentioning that despite PB seemingly failing to describe TAL-DNA binding free energy, it might actually capture important features of TAL recognition. It is in fact well known that TAL efficiently binds DNA only when the number of repeats is greater than 10.5. This behaviour is qualitatively described only by PB calculations, while GB energies monotonically decreases with the number of repeats.

Quantum mechanical calculations

Quantum Mechanical (QM) calculations were employed to obtain the molecular electrostatic potential (MEP) of DNA bases. The Gaussian 09 suite of program [19] was used to perform geometry optimisation and MEP calculation of nucleobases at the B3LYP/6-31+G*//B3LYP/6-31+G* level.

Methyl caps were added on the N9 of purines bases and the N1 of pyrimidine bases. The resulting MEP maps can be found in Figure 4B of the main text.

Pharmacophore-like model generation

The pharmacophore-like model of DNA nucleobases is a simplified 2D model suited to describe natural and modified purines and pyrimidines. Structural superposition of natural bases allowed defining a limited number of positions (loci), which vary across the various nucleobases and would interact differently with other molecules; the side-chain of X13 for the case of TAL proteins. Loci are colored according to their properties: green for pyridine-like (H-bond acceptor) nitrogen atoms, blue for pyrrole-like and amine (H-bond donor) nitrogen atoms, grey for methyl groups, and red for carbonyl oxygen atoms. The model can be found in Figure 4 of the main text.

Improving guanosine targeting

Molecular modeling was used to design new RVD constructs with improved selectivity for guanosine (with respect to NN) and ameliorated effectiveness (with respect to NK). A snapshot taken from the equilibrated part of the TAL[22.5]/P1 simulation was used as the starting geometry. Mutations/deletions were performed on repeat 16 (containing the RVD NN and targeting guanosine). The initial geometry was modified by *i)* mutating N13 into K (N12-N13K) or *ii)* removing G14 and mutating N13 into K (N13K-G14*) or *iii)* removing N13 residue and mutating G14 into K (N13*-G14K). Each mutated system was subjected to 10'000 steps of conjugate gradient energy minimization (as implemented in NAMD 2.8) to allow the rearrangement of the lysine side-chain at the protein-DNA interface. The full system was kept frozen during geometry optimization apart from residues within a 10 Å radius from the lysine C_α. The resulting geometries were visually inspected to evaluate the electrostatic and steric interactions between the DNA and the mutated TAL repeat. Mutating N13 into K (N12-N13K) caused a string deformation of both RVD loop and DNA leading strand in order to accommodate the longer side-chain of lysine. When the mutation was accompanied by deletion of one nearby residue, the shortened RVD loop allowed for a better accommodation of the lysine side-chain, resulting in a negligible deformation of the DNA strand while preserving a good interaction between the guanine and the amine moiety of lysine side-chain. Snapshots of the resulting structures can be found in Figure 5 of the main text.

Methylation

A modified protocol was used to estimate the effect of C5 cytosine methylation on TAL binding energies. An ensemble of 500 structures taken from the equilibrated part of the TAL[22.5]/P1 simulation (wildtype, *wt*) was used for the calculations. Two mutated systems were then constructed by modifying (in the selected snapshot of the *wt* simulation) the cytosine targeted by repeat 7 (RVD = N*): *i)* cytosine was methylated at C5 position or *ii)* the C-G base pair was mutated into a T-A base pair. The snapshots for the wildtype, the methylated and the mutated systems were subjected to a local minimization of 1'000 steps (conjugate gradient). The full protein-DNA complex was kept fixed

during minimization except for the base pair under investigation and its neighbours (*i-1* and *i+1*) and the closest protein residues (D13 from repeat 8 and the side-chain of N12 from repeat 6). MM/PBSA and MM/GBSA calculations were then performed on a trajectory reconstructed from the minimized snapshots. Bonded MM parameters for 5-methylcytidine were derived from those of cytidine and thymidine (AMBER ff99brsc) [5], charges for the phosphate and deoxyribose groups were taken from cytidine (AMBER ff99brsc) [5], while point charges of the methylcytosine (base) were obtained by RESP calculation at the HF/6-31g*//B3LYP/6-3+11g* on a 1,5-dimethylcytosine (4-amino-1,5-dimethylpyrimidin-2(1*H*)-one). The results of this analysis are summarized in Table 2 of the main text.

Supporting References

1. Levitt M (2001) The birth of computational structural biology. Nat Struct Biol 8: 392-393.
2. Karplus M, McCammon JA (2002) Molecular dynamics simulations of biomolecules. Nat Struct Biol 9: 646-652.
3. Berman HM, Westbrook J, Feng Z, Gilliland G, Bhat TN, et al. (2000) The Protein Data Bank. Nucleic Acids Res 28: 235-242.
4. Phillips JC, Braun R, Wang W, Gumbart J, Tajkhorshid E, et al. (2005) Scalable molecular dynamics with NAMD. J Comput Chem 26: 1781-1802.
5. Perez A, Marchan I, Svozil D, Sponer J, Cheatham TE, 3rd, et al. (2007) Refinement of the AMBER force field for nucleic acids: improving the description of alpha/gamma conformers. Biophys J 92: 3817-3829.
6. Jorgensen WL, Chandrasekhar J, Madura JD, Impey RW, Klein ML (1983) Comparison of Simple Potential Functions for Simulating Liquid Water. J Chem Phys 79: 926-935.
7. Bakan A, Meireles LM, Bahar I (2011) ProDy: protein dynamics inferred from theory and experiments. Bioinformatics 27: 1575-1577.
8. Humphrey W, Dalke A, Schulten K (1996) VMD: visual molecular dynamics. J Mol Graph 14: 33-38, 27-38.
9. Hunter JD (2007) Matplotlib: A 2D graphics environment. Comput Sci Eng 9: 90-95.
10. Mak ANS, Bradley P, Cernadas RA, Bogdanove AJ, Stoddard BL (2012) The Crystal Structure of TAL Effector PthXo1 Bound to Its DNA Target. Science 335: 716-719.
11. Consortium U (2012) Reorganizing the protein space at the Universal Protein Resource (UniProt). Nucleic Acids Res 40: D71-D75.
12. Deng D, Yan CY, Pan XJ, Mahfouz M, Wang JW, et al. (2012) Structural Basis for Sequence-Specific Recognition of DNA by TAL Effectors. Science 335: 720-723.
13. Kollman PA, Massova I, Reyes C, Kuhn B, Huo S, et al. (2000) Calculating structures and free energies of complex molecules: combining molecular mechanics and continuum models. Acc Chem Res 33: 889-897.
14. Rucker R, Oelschlaeger P, Warshel A (2010) A binding free energy decomposition approach for accurate calculations of the fidelity of DNA polymerases. Proteins 78: 671-680.
15. Noskov SY, Lim C (2001) Free energy decomposition of protein-protein interactions. Biophys J 81: 737-750.
16. Case DA, Cheatham TE, 3rd, Darden T, Gohlke H, Luo R, et al. (2005) The Amber biomolecular simulation programs. J Comput Chem 26: 1668-1688.
17. Xu L, Sun H, Li Y, Wang J, Hou T (2013) Assessing the Performance of MM/PBSA and MM/GBSA Methods. 3. The Impact of Force Fields and Ligand Charge Models. J Phys Chem B 117: 8408-8421.
18. Kuczera K, Kursula P (2012) Interactions of calmodulin with death-associated protein kinase peptides: experimental and modeling studies. J Biomol Struct Dyn 30: 45-61.
19. Frisch MJ, Trucks GW, Schlegel HB, Scuseria GE, Robb MA, et al. (2009) Gaussian 09 , Revision A.1. Wallingford CT: Gaussian 09, Revision A.1, Gaussian, Inc.
